# Dynamic Distribution of Linker Histone H1.5 in Cellular Differentiation

**DOI:** 10.1371/journal.pgen.1002879

**Published:** 2012-08-30

**Authors:** Jing-Yu Li, Michaela Patterson, Hanna K. A. Mikkola, William E. Lowry, Siavash K. Kurdistani

**Affiliations:** 1 Department of Biological Chemistry, David Geffen School of Medicine, University of California Los Angeles, Los Angeles, California, United States of America; 2 Eli and Edythe Broad Centre of Regenerative Medicine and Stem Cell Research, University of California Los Angeles, Los Angeles, California, United States of America; 3 Department of Molecular, Cellular, and Developmental Biology, University of California Los Angeles, Los Angeles, California, United States of America; 4 Department of Pathology and Laboratory Medicine, David Geffen School of Medicine, University of California Los Angeles, Los Angeles, California, United States of America; University of California San Francisco, United States of America

## Abstract

Linker histones are essential components of chromatin, but the distributions and functions of many during cellular differentiation are not well understood. Here, we show that H1.5 binds to genic and intergenic regions, forming blocks of enrichment, in differentiated human cells from all three embryonic germ layers but not in embryonic stem cells. In differentiated cells, H1.5, but not H1.3, binds preferentially to genes that encode membrane and membrane-related proteins. Strikingly, 37% of H1.5 target genes belong to gene family clusters, groups of homologous genes that are located in proximity to each other on chromosomes. H1.5 binding is associated with gene repression and is required for SIRT1 binding, H3K9me2 enrichment, and chromatin compaction. Depletion of H1.5 results in loss of SIRT1 and H3K9me2, increased chromatin accessibility, deregulation of gene expression, and decreased cell growth. Our data reveal for the first time a specific and novel function for linker histone subtype H1.5 in maintenance of condensed chromatin at defined gene families in differentiated human cells.

## Author Summary

In human cells, there are eleven subtypes of linker histones, five (H1.1–H1.5) of which are ubiquitously expressed in somatic cells. Somatic linker histones have been thought of as a group of similar proteins with redundant functions with few known differences among them. Our work uncovers for the first time a novel and unique role for the linker histone H1.5 (HIST1H1B). We found that H1.5, but not H1.3 (HIST1H1D), forms blocks of chromatin binding in genic and intergenic regions in differentiated human cells from all germ layers but not in embryonic stem cells. In genic regions, H1.5 binds to a large fraction of gene families that encode membrane associated proteins and are transcriptionally silent in a tissue-specific manner. H1.5 binding is associated with other repressive chromatin elements such as SIRT1 binding and H3K9me2 enrichment, and it negatively correlates with Pol II distribution. SIRT1 and H3K9me2 binding is dependent on H1.5, but not vice versa. H1.5 depletion in fibroblasts leads to increased chromatin accessibility at its target loci, altered cell cycle, and deregulation of gene expression. Our findings show that H1.5 has a dynamic distribution during human cell differentiation and is required for maintenance of proper gene expression in differentiated cells.

## Introduction

In humans, there are eleven subtypes of linker histones that stabilize higher order chromatin structure and are generally associated with repressed genes 1–5. Depletion of mouse H1c, H1d and H1e leads to less compact packaging of chromatin, changes in core histone modifications, and reduced DNA methylation at certain loci [6]. Binding of H1 and poly (ADP-ribose) polymerase-1 at 758 RNA polymerase II (Pol II)-transcribed promoters is mutually exclusive at actively transcribed genes [7]. In human cancer, linker histones exhibit altered expression with at least one linker histone gene, namely H1.5, being mutated in colon cancer [8]. Linker histones are, therefore, important participants in normal biological as well as disease processes. However, while some functional differences have been reported for certain linker histones [9], our knowledge of global distribution or function of each linker histone remains rudimentary.

Gene families are groups of homologous genes that are likely to have highly similar functions. While some gene family members are dispersed throughout the genome (e.g., solute carrier protein genes or SLCs), others are located in close physical proximity to each other, forming clusters of functionally related genes on human chromosomes. These gene family clusters include the olfactory receptor (OR), late cornified envelope (LCE), histone (HIST) and homeobox (HOX) genes. Current data indicate that different gene families have distinct chromatin features. For instance, the chromatin regions of OR and certain other gene family clusters lack histone modifications such as histone H3 lysine 4 methylation (H3K4me) and H3K27me that are found in the HOX clusters [10], [11]. Considering the diversity of gene families in the human genome, it is not expected *a priori* that they would share similar chromatin characteristics or regulatory mechanisms.

Here we show for the first time that human linker histone H1.5 (HIST1H1B) binds to families of genes that are enriched for those encoding membrane or membrane-related proteins in terminally differentiated cell types representing all three embryonic germ layers. Little or no H1.5 enrichment was detected at the majority of the gene families in undifferentiated human embryonic stem cells (hESCs). H1.5 interacts with SIRT1 histone deacetylase which, along with H3K9me2, a repressive histone modification, were also enriched at H1.5 targets. Furthermore, H1.5 bound regions were mutually exclusive of DNase I sensitive regions. H1.5 depletion in fibroblasts resulted in disturbed SIRT1 and H3K9me2 distribution, and decreased chromatin compaction specifically at target genes. H1.5 knockdown cells showed extensive global deregulation of gene expression, with de-repression of certain H1.5 target genes. Together, our findings reveal an unexpected but widespread function of histone H1.5 in chromatin compaction and gene expression in differentiated human cells.

## Results

### Histone H1.5 is differentially distributed in hESCs and fibroblasts

**Figure 1:**
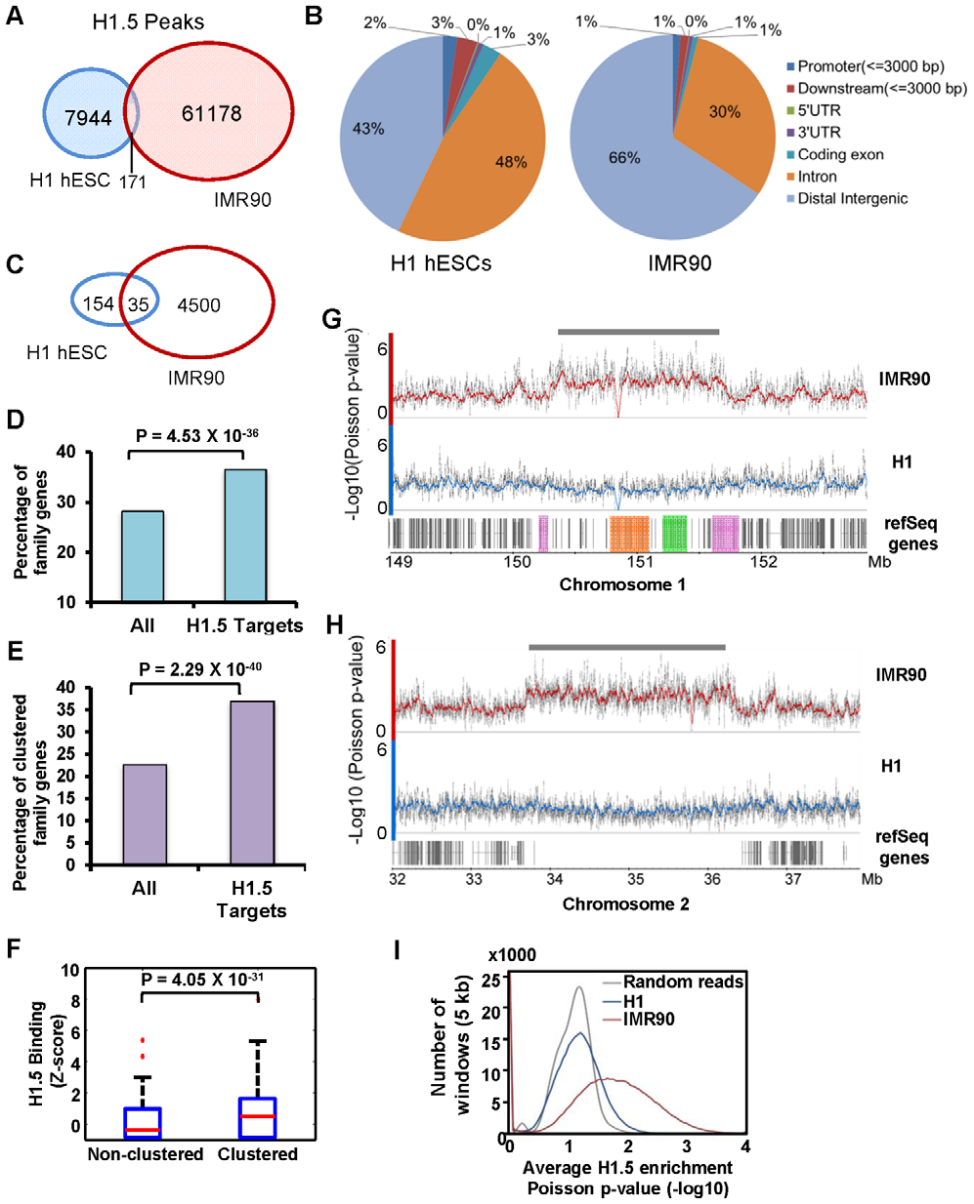
Histone H1.5 is differentially distributed in fibroblasts and embryonic stem cells. (A) Venn diagram of significant peaks of H1.5 binding in H1 hESCs and IMR90 fibroblasts by ChIP-seq. (B) Pie chart of distribution of H1.5 relative to gene structure in H1 hESCs and IMR90 fibroblasts. (C) Venn diagram of number of H1.5 target genes in H1 hESCs and IMR90 fibroblasts. (D) Enrichment of HGNC gene family members in H1.5 target genes. Height of bars represents percentage of gene family members in all RefSeq genes (left) or H1.5 target genes (right). (E) Enrichment of clustered gene family members in H1.5 target genes. Height of bars represents percentage of clustered gene family members in all RefSeq genes (left) or H1.5 target genes (right). (F) Box plot of H1.5 enrichment levels of non-clustered gene family members (left) and clustered gene family members (right). (G) H1.5 enrichment block at the LCE/SPRR/S100A gene clusters. Each dot represents −Log10 of Poisson p-value of ChIPed DNA versus input DNA in a 100-bp window. Lines represent average values. LCE, SPRR and S100A genes are highlighted in orange, green and pink, respectively. (H) An H1.5 enrichment block in an intergenic region of chromosome 2. (I) Histogram of average significance of H1.5 enrichment (x-axis) in 5 kb windows versus the number of windows (y-axis) in H1 hESCs and IMR90 fibroblasts.

To determine whether the genomic distribution of H1.5 is different in hESCs versus fibroblasts, we used chromatin immunoprecipitation combined with sequencing (ChIP-seq) of linker histone H1.5 in H1 hESCs and human lung IMR90 fibroblasts. We defined a significant peak as enrichment of ChIP over input DNA within a 100-bp window at a Poisson p-value<0.001. We detected 8115 and 61349 significant peaks of H1.5 in H1 hESCs and IMR90, respectively, with only 171 peaks shared between the two cell lines (Figure 1A). In H1 hESCs, the peaks of H1.5 were distributed between genic regions (±3 kb of genes) and distal intergenic regions which are at least 3 kb away from any gene. In IMR90 cells, the majority of H1.5 peaks were in distal intergenic regions with the rest within genic regions (Figure 1B).

To understand the genome-wide distribution of H1.5, we calculated H1.5 peak density (number of peaks per kb of genomic DNA) in genic regions, which we defined as −3 kb from annotated transcription start sites (TSS) to +3 kb from annotated transcriptional stop sites (TTS), as well as intergenic regions. Regions with at least one significant peak (see Text S1) per kb were defined as H1.5 target loci. Genes that were bound by H1.5 in their genic regions or were located upstream or downstream of intergenic regions bound by H1.5 were defined as H1.5 target genes. In IMR90 fibroblasts, we detected 4535 H1.5 target genes with 1204 genes directly bound by H1.5, 2294 genes next to H1.5 target intergenic regions, and 1037 genes bound by H1.5 in both genic and neighbouring intergenic regions (Figure S1A). In hESCs, only 189 H1.5 target genes were detected (Figure 1C). Gene ontology analysis showed no significant enrichment for hESCs but enrichment of H1.5 target genes in membrane associated receptor and signalling genes in fibroblasts (Table 1). Among the 4535 genes bound by H1.5 in IMR90, we noticed that some of them belonged to gene families, such as olfactory receptor family and other G-protein coupled receptors, and solute carrier family. This prompted us to systematically determine the enrichment of H1.5 target genes within the HGNC (HUGO Gene Nomenclature Committee) gene family database. Of the 4535 H1.5 target genes, 1659 (37%) genes were members of gene families, whereas all gene family members only accounted for 28% of total genes in human genome (binomial p-value = 4.53E-36), indicating a significant enrichment of gene families within H1.5 targets (Figure 1D). The genes from the same family can be either scattered throughout the genome or clustered in close physical proximity. When considering at least three genes from the same family that are located side by side as a ‘cluster’, ∼23% of genes from HGNC gene families form clusters throughout the genome. Interestingly, the percentage of clustered genes increased to 37% within H1.5 target genes (Figure 1E; binomial p-value = 2.29E-40). In addition, the level of H1.5 binding at clustered genes is significantly higher than that in non-clustered genes (Figure 1F). Figure 1G shows an example of H1.5 binding to a block of ∼1.5 Mbp on chromosome 1 covering the S100 calcium binding protein A (S100A), LCE, and small proline-rich (SPRR) gene family clusters in IMR90 cells, but not in H1 hESCs. This H1.5 binding block also includes fifteen genes that do not belong to a gene family but are located in between the three gene family clusters. Interestingly, five of these fifteen genes are involved in “keratinocytes differentiation” (binomial p-value = 2.2E-06) and two are peptidoglycan recognition proteins, suggesting functional relatedness to the nearby gene families.

**Table 1:** Gene ontology of H1.5 target genes in IMR90 fibroblasts.

Biological Process	P-Value	FDR
GO:0007186 G-protein coupled receptor protein signaling pathway	1.59E-35	2.85E-32
GO:0050877 neurological system process	1.17E-28	2.10E-25
GO:0007600 sensory perception	2.30E-26	4.13E-23
GO:0007166 cell surface receptor linked signal transduction	3.43E-26	6.15E-23
GO:0050890 cognition	5.34E-26	9.58E-23
GO:0006952 defense response	1.11E-08	2.00E-05
GO:0007155 cell adhesion	1.14E-08	2.05E-05
GO:0007267 cell-cell signaling	3.42E-07	6.13E-04

Cellular Component	P-Value	FDR
GO:0005886 plasma membrane	1.53E-29	2.14E-26
GO:0031224 intrinsic to membrane	2.57E-20	3.58E-17
GO:0016021 integral to membrane	1.91E-18	2.66E-15
GO:0005576 extracellular region	2.37E-14	3.31E-11

Blocks of H1.5 enrichment were also found in intergenic regions in IMR90 cells but not in hESCs (Figure 1H). To determine if this block pattern is a genome-wide feature of H1.5 binding in fibroblasts, we calculated the average significance of H1.5 peaks within 5 kb windows and plotted the number of windows as a function of average enrichment p-value (Figure 1I). Compared to a random set of peaks (grey line) or the H1.5 peaks in hESCs (blue line), in IMR90 cells there were many windows with no significant enrichment of H1.5 but also many more windows with highly significant enrichment (red line). This distribution indicates that H1.5 forms blocks of enrichment over both genic and intergenic regions in IMR90 cells but not in hESCs. Remarkably, H1.5 genic blocks of enrichment occurs preferentially at a subset of gene families.

**Figure 2:**
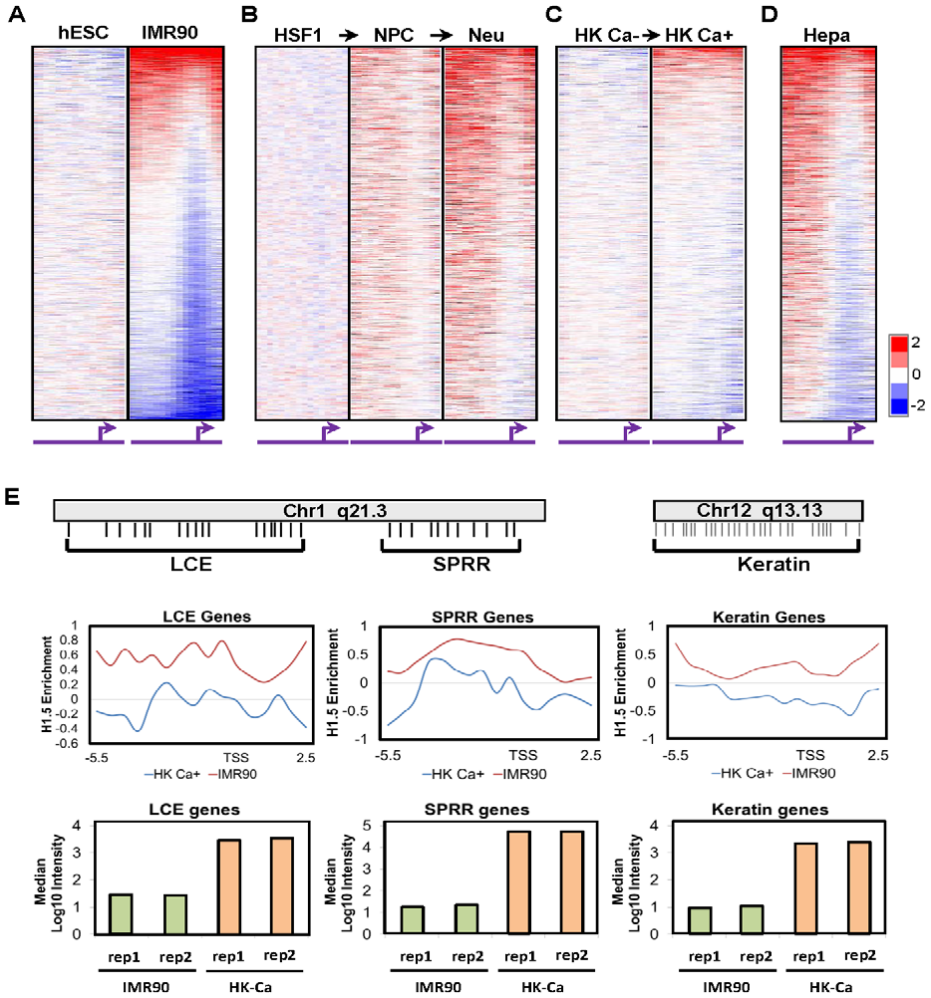
H1.5 distribution is established during cellular differentiation. Heat maps show the genome wide promoter distribution of H1.5 in (A) H1 hESCs and IMR90 fibroblasts, (B) HSF1 hESCs, neural progenitor cells (NPC), neural cells (Neu), and (C) primary keratinocytes (HK Ca−), calcium-treated keratinocytes (HK Ca+), and (D) primary human hepatocytes (Hepa). Each row represents the promoter of a gene in 500-bp intervals from −5.5 to +2.5 kb of the transcription start sites (TSS) which is indicated by the arrows. The gene promoters for all heat maps are ordered based on the highest to lowest level of H1.5 enrichment in fibroblasts. (E) Average H1.5 enrichment and expression of the epidermal (LCE, SPRR) and KRT gene clusters in HK Ca+ and IMR90 fibroblasts are shown as line charts and bar graphs, respectively. The relative position of genes in each cluster is illustrated schematically.

To confirm the H1.5 enrichment patterns in IMR90 fibroblasts, we used an Agilent promoter microarray containing probes for ∼17,000 gene promoters, tiling an 8-kb region from −5.5 to +2.5 kb of the annotated transcriptional start sites (TSS) which we divided computationally into 16 fragments of 500 bp each (Figure S1B). We first verified the specificity of the H1.5 ChIP signal by ChIP-chip analysis of H1.5 after knockdown (KD) of H1.5 in IMR90 cells, which showed loss of H1.5 signal and no preferential enrichment compared to the control KD (Figure S1C). Consistent with ChIP-seq data, ChIP-chip of H1.5 showed significantly more enrichment in fibroblasts (Figure 2A; gene promoters are ranked from highest to lowest H1.5 enrichment in fibroblasts; all other ChIP-chip heat maps are in the same order). Gene ontology analysis of fibroblast data revealed that 59% of genes bound by H1.5 on the array were members of HGNC gene families (binomial p-value = 1.4E-08). When we sorted the genes based on their locations on each chromosome, we found that H1.5 was enriched in blocks of consecutive promoters (Figure S1D). For instance, on chromosome 1, the promoter blocks comprised families of highly homologous genes including LCE, SPRR, Fc receptor-like (FCRL) and the OR genes. Similarly, the promoter blocks on chromosome 11 corresponded to several OR gene clusters. This binding pattern was specific to H1.5 as H1.3 (HIST1H1D) did not show preferential binding to the gene families in IMR90 cells (Figure S1D). ChIP-quantitative PCR (qPCR) of selected genes confirmed the preferential binding of H1.5 to the gene families in IMR90 cells (Figure S2). Moreover, binding of H1.5 is not related to increased nucleosome density as histone H3 ChIP-chip did not show significant enrichment at H1.5 target genes in hESCs or fibroblasts (Figure S1E).

### H1.5 binding is dependent on cellular differentiation state

Since hESCs and fibroblasts represent the extremes of cellular differentiation states, we sought to determine the differentiation stage at which the binding pattern of H1.5 is established. We examined H1.5 distribution in two cellular differentiation systems. First, we specified HSF1 hESCs to neural progenitor cells (NPCs) and then used standard growth factor withdrawal to drive differentiation towards neurons and astrocytes [12] (Figure 2B; see Methods). Interestingly, H1.5 binding was established in terminally differentiated neural cells with the NPCs showing an intermediate pattern of H1.5 binding. Second, we obtained primary keratinocytes from skin biopsies and induced them to further differentiate *in vitro* using calcium (Ca^2+^) which promotes primary keratinocytes to exit cell cycle and form stratified layers in culture [13]. Like the neural differentiation, H1.5 binding was more significant in more differentiated, Ca^2+^-treated keratinocytes (Figure 2C). Finally, we also examined H1.5 binding pattern in primary hepatocytes (Figure S3), which are derived from endoderm, and found similar H1.5 binding pattern (Figure 2D) as in fibroblasts and neural cells which are derived from mesoderm and ectoderm, respectively. Altogether, these data indicate that the H1.5 binding pattern is established progressively as cells acquire a more differentiated phenotype and occurs in fully differentiated cells derived from all three embryonic germ layers.

### H1.5 binding pattern is tissue specific

**Figure 3:**
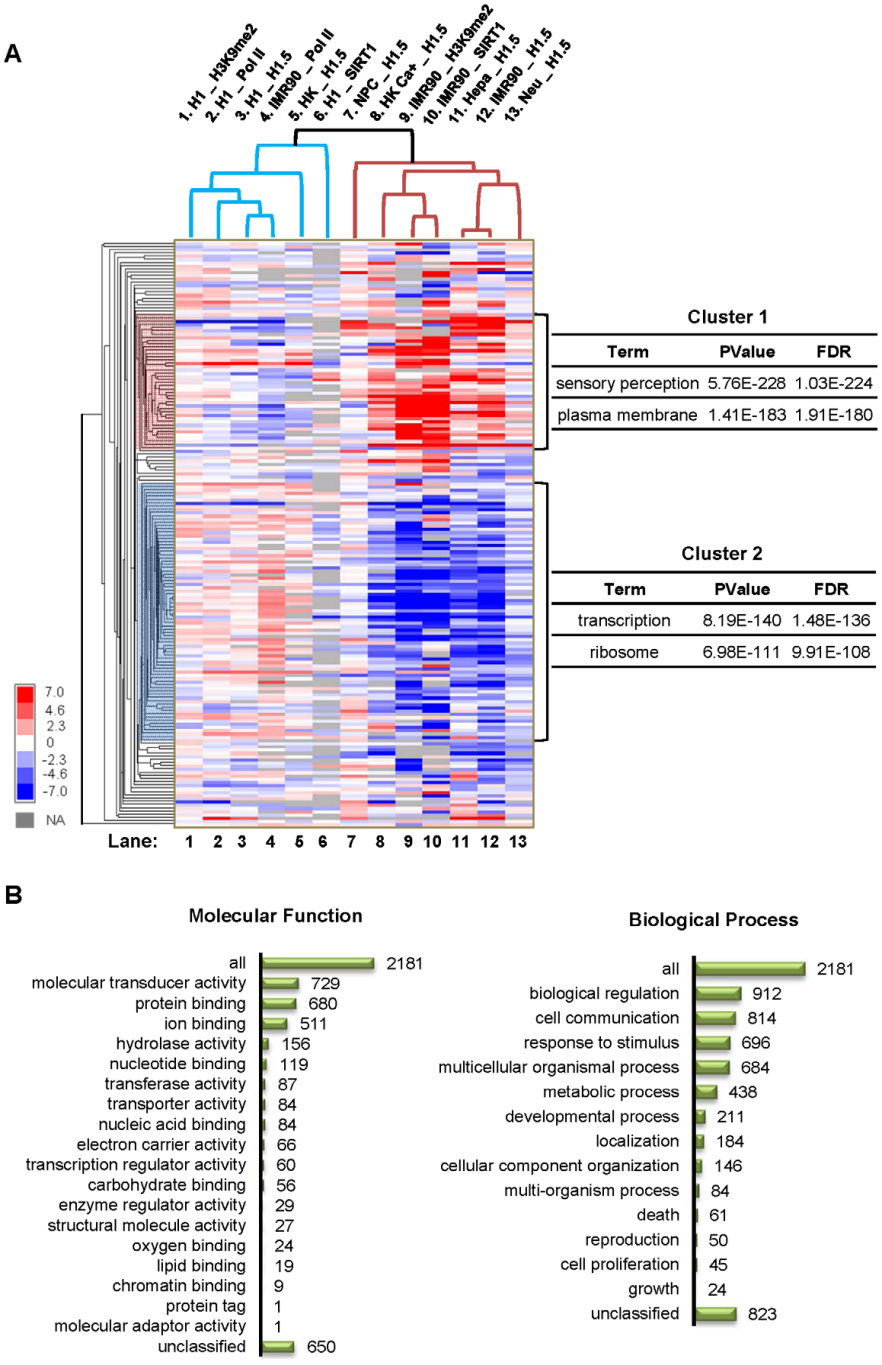
H1.5 enrichment in HGNC gene families. (A) Hierarchical clustering of H1.5 enrichment levels in HGNC gene families is shown as a heat map. Enrichment z-score of each gene family was calculated by averaging the intensities of probes within a gene family region corrected for number of probes. Each row represents a gene family, and each column represents an experiment. The main sub-clusters of gene families are highlighted on the left and the most enriched gene ontology terms for each cluster are shown on the right. (B) Ontology classification of genes in cluster 1 families. Number of genes in each category is indicated.

Despite the similarity of H1.5 binding patterns in different cell types, we noticed some degree of tissue specificity (Figure 3). For instance, LCE and SPRR genes form an “epidermal gene cluster”, which together with the keratin gene cluster (Figure 2E scheme), are highly expressed in all keratinocytes (with or without Ca^2+^ treatment) compared to IMR90 fibroblasts (Figure 2E bar charts). The enrichment of H1.5 specifically at the LCE, SPRR and KRT gene clusters in keratinocytes is significantly lower than that of IMR90 fibroblasts (Figure 2E line chart) or other cell types (Figure 3A, see bleow). These data suggest that the histone H1.5 binding pattern in differentiated cells is tissue specific, with H1.5 being depleted from gene families that are expressed appropriately in certain cell types.

### H1.5 binds to specific gene families

To systematically study H1.5 enrichment in gene families, we generated a matrix containing enrichment values of H1.5 (as well as RNA Pol II, SIRT1, and H3K9me2; see below) in 188 HGNC gene families across all cell types analysed in this study. As shown in figure 3A, the enrichment values were clustered hierarchically across cell types/experiments (columns) and gene families (rows). Interestingly, the main branch point in the columns separated the differentiated cells with a dynamic pattern of H1.5 enrichment (Figure 3A, Lanes 7–13) from those with little or no preferential enrichment of H1.5 (Figure 3A, Lanes 1–6). The gene families (rows) were grouped into two main sub-clusters. Cluster 1 included gene families that were bound by H1.5 in at least two of the differentiated cell lines. Gene ontology analysis of genes in cluster 1 families revealed highly significant enrichment for membrane-associated proteins and sensory perception (Figure 3A). In contrast, cluster 2 gene families that were depleted of H1.5 were significantly enriched for ribosome associated proteins and those involved in transcription regulation (Figure 3A). By classifying the 2181 genes in cluster 1 gene families based on their molecular function or biological process [14], we detected 729 genes (p = 1.17E-256) as signal transducers, 657 of which have receptor activity (p = 2.71E-264). 696 genes are involved in stimulus response (p = 6.81E-94) and 912 genes are involved in biological regulation (p = 1.68E-13), in which 666 genes play roles in cell surface receptor linked signal transduction (p = 2.96E-222) (Figure 3B). These data indicate that H1.5 preferentially binds to a defined subset of membrane and membrane-associated gene families in differentiated cells.

### Binding of H1.5 is associated with repressed genes

**Figure 4:**
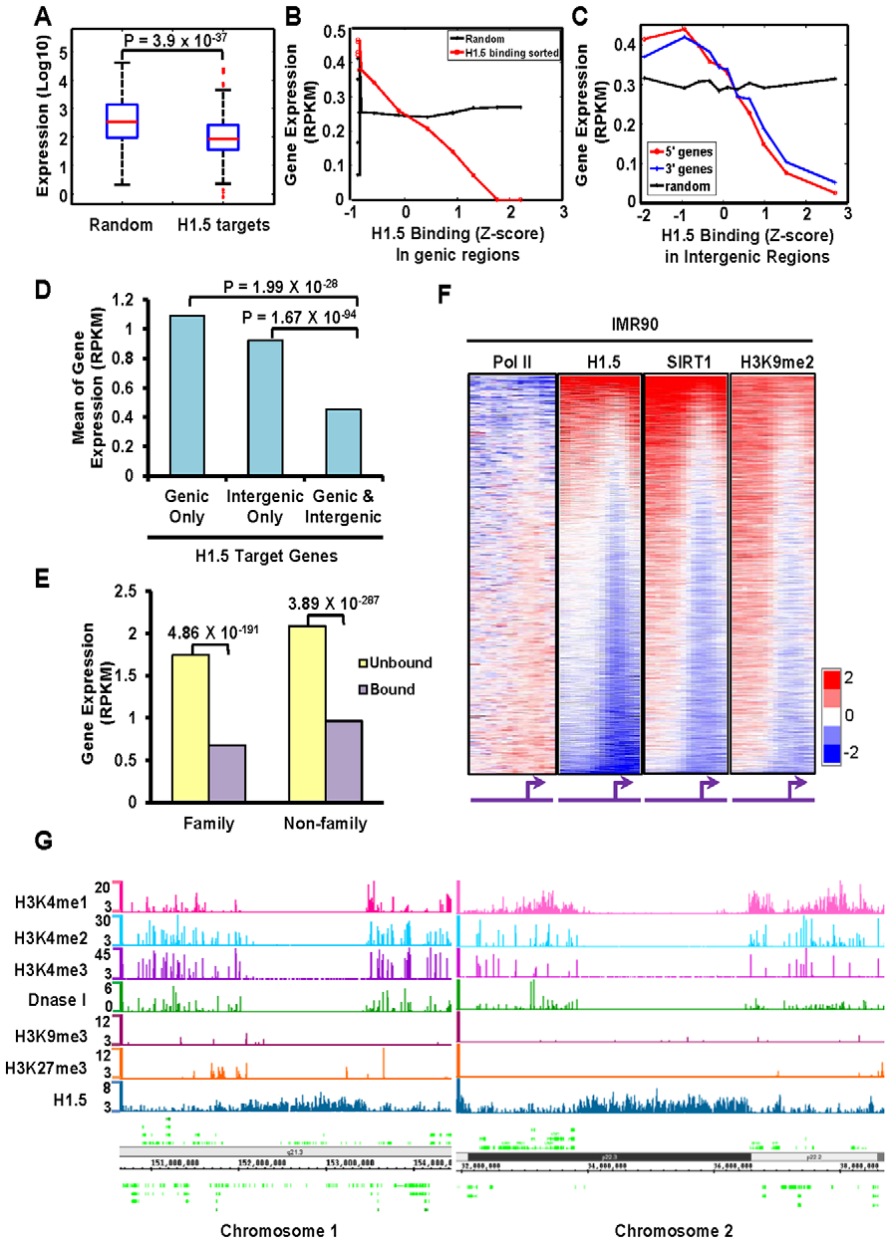
H1.5 is associated with repressed genes. (A) Boxplots of expression levels (Affymetrix array) of randomly selected genes (left) and H1.5 target genes (right) in IMR90 fibroblasts. (B–C) Line charts show gene expression levels (mRNA-seq) as a function of H1.5 binding at (B) genic regions or (C) intergenic regions in control knockdown IMR90 fibroblasts. All RefSeq genes were sorted by H1.5 binding levels; each data point represents the average expression value of 2000 genes. Genes located upstream or downstream of an intergenic region were denoted as 5′ gene (red line) and 3′ gene (blue line), respectively. (D) Average expression level of genes bound by H1.5 at either genic (left), intergenic (middle), or both (right) regions in IMR90 fibroblasts. Wilcoxon rank sum test p-values are indicated. (E) Average expression level of genes that belong (‘family’) or do not belong (‘non-family’) to HGNC gene families that were bound (purple bars) or not bound (yellow bars) by H1.5 in IMR90 fibroblasts. (F) Genome-wide promoter binding of Pol II, H1.5, SIRT1 and H3K9me2 in IMR90 fibroblasts. (G) Distributions of H3K4me1, H3K4me2, H3K4me3, DNase I sensitive sites, H3K9me3, H3K27me3, and H1.5 peaks at LCE/SPRR/S100A gene cluster (left panel) and an intergenic region in chromosome 2 (right panel). The scale of DNase I hypersensitive sites represent z-score of counts in each 100-bp window. Scales of H1.5 and other histone modifications represent the Poisson p-values of enrichment at each 100-bp window.

To determine whether H1.5 is associated with transcriptional repression, we examined the global gene expression profile in IMR90 cells, hepatocytes, HK Ca+ and hESCs. The expression level of H1.5 target genes were significantly lower than that of a randomly-selected, similarly-sized group of genes in the three differentiated cell types but not in hESCs (Figure 4A and Figure S4A–S4C). To further characterize the association between H1.5 binding and transcription, we sequenced messenger RNAs (mRNAs) from IMR90 fibroblasts (transfected with non-targeting siRNAs which will be later used as control for H1.5 knockdown cells), and compared the expression to H1.5 binding. As shown in Figure 4B, when we sorted all RefSeq genes by H1.5 enrichment and divided these genes into 11 groups (2,000 genes per group), the average gene expression level in each group decreased with increasing H1.5 binding (r = −0.39). Interestingly, H1.5 binding level in intergenic regions was also negatively correlated with the expression of neighbouring genes (r = −0.17 for 5′ genes and r = −0.14 for 3′ genes, Figure 4C). Genes that were bound by H1.5 in their genic and intergenic regions were more significantly repressed than those that were bound at either region (Figure 1D). Within the H1.5 target genes, both those belonging to families and non-families were equally repressed (Figure 4E). In addition, we also examined Pol II binding which was negatively correlated with H1.5 (r = −0.19; Figure 4F). Pol II binding at the gene families also showed an opposite pattern to that of H1.5 (Figure 3A, compare lanes 4 and 12). Taken together, we conclude that the binding of linker histone H1.5 is correlated with depletion of Pol II and repression of target genes in differentiated cells.

### SIRT1 and H3K9me2 bind to H1.5 target genes

Vaquero et al. reported previously that human SIRT1 interacts with linker histone subtype HIST1H1E [15]. Considering the high amino acid sequence conservation between HIST1H1E and H1.5, we asked whether H1.5 also interacts with SIRT1, and if so, whether the genomic distributions of SIRT1 and H1.5 overlap. Reciprocal co-immunoprecipitation experiments from IMR90 and hESC nuclear extracts revealed a direct or indirect H1.5-SIRT1 interaction in IMR90 cells, but not in hESCs (Figure S4). Consistently, the SIRT1 binding pattern at promoter regions in fibroblasts was highly similar to that of H1.5 (Figure 4F, r = 0.58). Furthermore, SIRT1 deacetylates H3K9 which then can serve as a substrate for methylation. H3K9 methylation has been shown to be enriched at repressed regions [16]. Thus, we examined H3K9me2 distribution at promoter regions in fibroblasts which was also very similar to H1.5 (r = 0.56) and SIRT1 (r = 0.67) binding (Figure 4F). Like H1.5, H3K9me2 and SIRT1 were also enriched in gene families involved in sensory perception, and clustered together with H1.5 enrichment in differentiated cells (Figure 3A, Lanes 9 and 10). These data suggest that H1.5, SIRT1 and H3K9me2 associate with defined gene sets that are normally repressed. Analyses of published data on distributions of other histone modifications including H3K4me1, H3K4me2, H3K4me3, H3K9me3 and H3K27me3 in IMR90 cells revealed little overlap with H1.5 binding at representative target gene cluster (Figure 4G; left panel) and intergenic regions (Figure 4G; right panel) or globally (Figure S5A–C).

### H1.5 is required for SIRT1 and H3K9me2 enrichment

**Figure 5:**
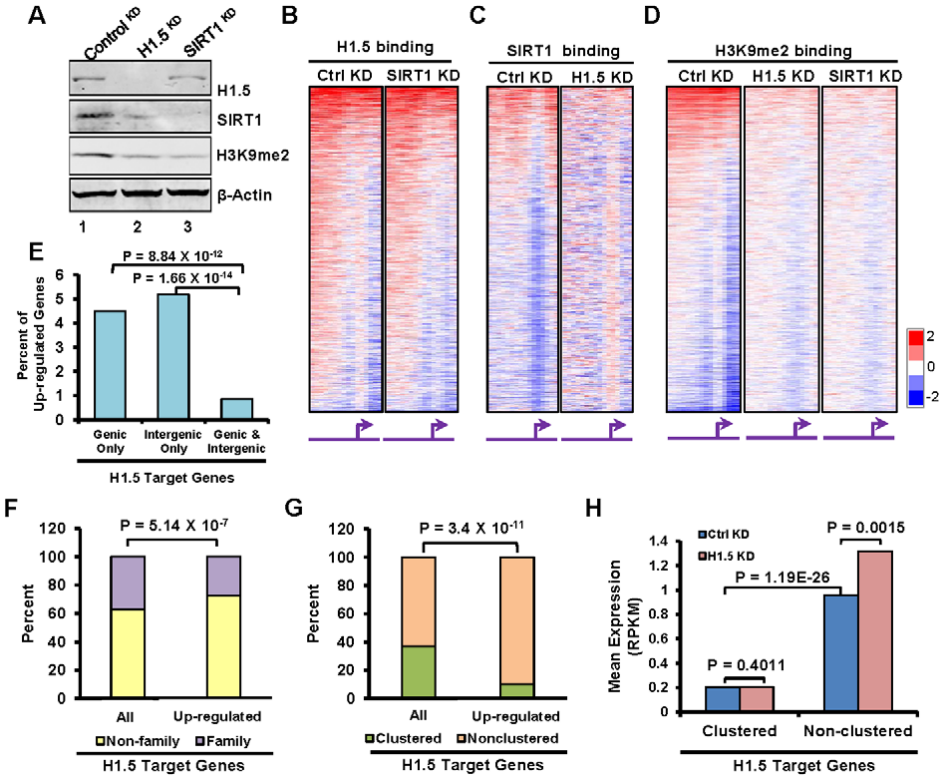
H1.5 is required for enrichment of SIRT1 and H3K9me2 at H1.5 target loci and normal gene expression pattern. (A) Western blotting of H1.5, SIRT1, H3K9me2, and β-Actin in control^KD^, H1.5^KD^, and SIRT1^KD^ IMR90 cells. (B–D) Genome wide promoter binding of the indicated factors and experimental conditions is shown as heat maps. The genes are ordered as in Figure 2A. (E) Percentage of up-regulated genes with the indicated H1.5 binding pattern. The binomial p-values are indicated. (F) Stacked bar chart of percentage of family (yellow) and non-family (purple) genes in all H1.5 targets (left bar) and up-regulated H1.5 targets (right bar). The binomial p-value is indicated. (G) Stacked bar chart of percentage of clustered (orange) and non-clustered (burlywood) in all H1.5 targets (left bar) and up-regulated H1.5 targets (right bar). The binomial p-value is indicated. (H) Expression levels (mRNA-seq) of clustered and non-clustered H1.5 target genes in control^KD^ and H1.5^KD^ cells are shown as a bar chart.

To determine whether SIRT1 and H1.5 regulate chromosomal distribution of each other, we transfected IMR90 cells with siRNAs to knockdown (^KD^) H1.5 or SIRT1 and mapped the binding of the other. Knockdown of H1.5 or SIRT1 did not affect the expression levels of other linker histone subtypes (Figure S6A). In H1.5^KD^ IMR90 cells (Figure 5A, lane 2), SIRT1 expression was down-regulated, and its distribution was globally disrupted (Figure 5C, r = 0.012). In contrast, H1.5 expression was not changed significantly by SIRT1 knockdown (Figure 5A, lane 3), and its distribution was only partially affected (Figure 5B, r = 0.47), indicating that H1.5 binding is less dependent on SIRT1. Knockdown of either protein resulted in lower H3K9me2 levels (Figure 5A) and loss of H3K9me2 enrichment (Figure 5D), suggesting that both proteins are required for establishment of this repressive histone mark.

### H1.5 knockdown leads to global deregulation of gene expression

To determine if depletion of H1.5 affects gene expression, we sequenced mRNAs from H1.5^KD^ and control^KD^ IMR90 fibroblasts. We detected 2367 genes with at least 1.5 fold change in expression in H1.5^KD^ versus control^KD^ cells, 2022 (85%) of which were up-regulated in H1.5^KD^ cells. Among the genes with deregulated gene expression, 371 genes were H1.5 targets, and 345 (93%) of them were up-regulated. Interestingly, many more genes were up-regulated if they were bound by H1.5 in either genic or intergenic regions than in both (Figure 5E), indicating that genes located in larger H1.5 binding blocks were less affected by H1.5 depletion (which could be due to incomplete KD of H1.5). Consistently, we found that over 70% of the up-regulated H1.5 target genes do not belong to gene families (Figure 5F), which have higher levels of H1.5 binding (Figure 1F). Finally, over 90% of up-regulated genes in H1.5 targets are non-clustered, which is significantly higher than the percentage of non-clustered genes in all H1.5 targets (Figure 5G). The expression level of non-clustered H1.5 target genes was significantly increased in H1.5^KD^, while clustered genes were not affected (Figure 5H). These data indicate that the singleton H1.5 target genes are more readily de-repressed in H1.5^KD^ cells. The lack of de-repression of H1.5-target clustered genes may be due to incomplete knockdown of H1.5, lack of appropriate transcriptional activators in fibroblasts or additional but unknown layers of gene regulation.

Overall, the up-regulated genes in H1.5^KD^ cells were enriched in cell death and apoptosis, whereas the down-regulated genes were enriched in DNA replication and cell cycle process (Figure S6B). Consistent with these changes, we found that knockdown of H1.5 significantly decreased the growth of cells as well as the proportion of cells in S and G2/M phases of the cell cycle (Figure S6C), suggesting that H1.5 is required for normal cell growth. Altogether, these data suggest that disruption of H1.5 affects the expression of >10% of all genes, contributing to altered cell cycle and growth of fibroblasts.

### H1.5 binding is required for chromatin compaction

**Figure 6:**
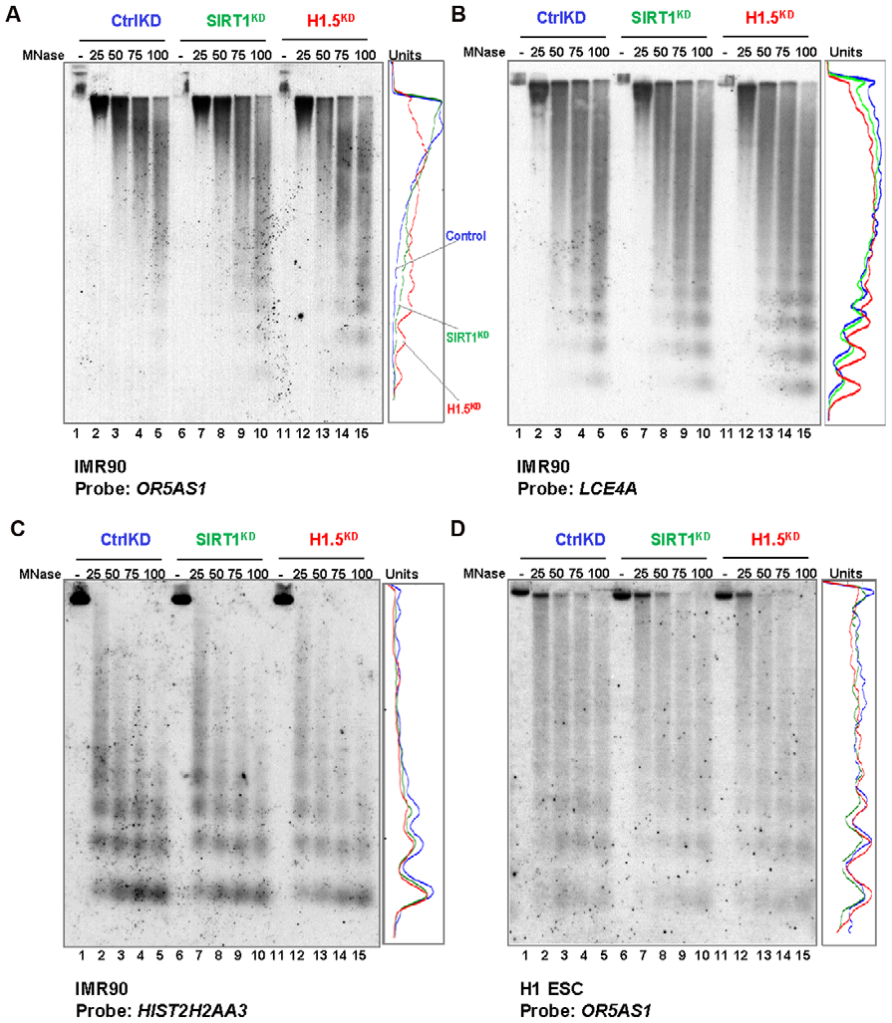
H1.5 knockdown increases micrococcal nuclease accessibility at target regions. (A–D) Southern blots of MNase digested genomic DNA form the indicated cells are shown. The probe used for each blot is also indicated. Quantitated data from lanes 5, 10, and 15 are shown as line chart. Y axis represents the pixel position in the images, and x axis shows the band intensity.

The formation of H1.5 enrichment blocks in IMR90 cells prompted us to ask whether H1.5 functions to compact chromatin at its target regions. We performed micrococcal nuclease (MNase) assays in control^KD^, SIRT1^KD^ and H1.5^KD^ IMR90 fibroblasts and H1 hESCs. Figure S7A and S7B show the ethidium bromide staining of the MNase digested DNA from the indicated conditions. To determine the accessibility at regions targeted by H1.5, we performed Southern blotting using a fragment of one H1.5 target gene *OR5AS1* (Figure S7C) on chromosome 11 as probe. The lanes corresponding to the highest concentrations of MNase were quantitated and visualized as line charts. As shown in Figure 6A, in H1.5^KD^ IMR90 cells nucleosomal DNA repeat length at the *OR5AS1* gene locus appeared earlier with increased intensity than control cells, indicating greater accessibility to MNase. Interestingly, SIRT1^KD^ cells showed an intermediate level of accessibility with greater digestion than control but less than H1.5^KD^ cells. A similar result was also detected when using *LCE4A* gene, another H1.5 target gene (Figure S7C), as a probe (Figure 6B). In contrast, H1.5^KD^ or SIRT1^KD^ IMR90 cells did not show MNase accessibility differences at a histone gene (*HIST2H2AA3*) locus that is not bound by H1.5 (Figure 6C). In H1.5^KD^ or SIRT1^KD^ H1 hESCs, we did not see significant differences in MNase digestion pattern of the *OR5AS1* gene locus compared to control^KD^ (Figure 6D). Therefore, H1.5 contributes to compaction of chromatin at its target loci.

**Figure 7:**
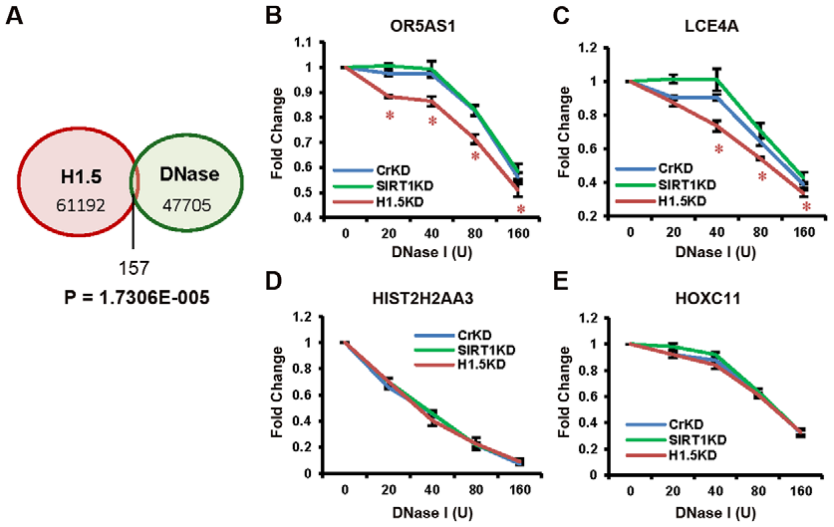
H1.5 knockdown increases DNase I sensitivity at target regions. (A) Venn diagram of the overlap between significant H1.5 peaks and DNase I hypersensitive sites. The p-value for exclusivity of these two sets of peaks is indicated (Text S1). (B–E) Quantitative PCR of DNA fragments at indicated genes from genomic DNA treated with increasing amount of DNase I. Data points with t-test p-value<0.05 are labelled with *.

Finally, we determined the relationship between H1.5 enrichment and chromatin accessibility by comparing our H1.5 ChIP-seq data with published DNase-seq data from IMR90 fibroblasts (GSM530665). Remarkably, H1.5 enriched regions were clearly excluded from DNase I sensitive regions with only 0.26% of H1.5 peaks overlapping with DNase I sensitive regions (Figure 7A; also see Figure 4G green lane). To determine if H1.5 knockdown increases DNase I sensitivity at target loci, we treated cell nuclei from control^KD^, SIRT1^KD^, and H1.5^KD^ IMR90 cells with increasing amount of DNase I followed by quantitative amplification of two H1.5 target genes (*OR5AS1* and *LCE4A*) and two non-target genes (*HIST2H2AA3* and *HOXC11*). In H1.5^KD^ cells, more digestion was detected at *OR5AS1* and *LCE4A* gene loci (Figure 7B and 7C) compared to control^KD^ and SIRT1^KD^ cells. At *HIST2H2AA3*, a potentially euchromatic locus, we did not observe significant differences in DNase I sensitivity between H1.5^KD^ and control^KD^ cells (Figure 7D). Importantly, *HOXC11* which is not a target of H1.5 but enriched for H3K27me3 (Figure S7C) also did not exhibit sensitivity to DNase I digestion (Figure 7E). These data indicate that H1.5 target regions are less accessible and knockdown of H1.5 primarily affects the chromatin compaction at its target regions.

## Discussion

Mammalian cells at different stages of differentiation are generally thought to have dissimilar chromatin structures [17], [18]. Our data indicate that the linker histone subtype H1.5 contributes to dynamic formation of compact blocks of chromatin in differentiated cells of all embryonic germ layers. These blocks were found in intergenic regions as well as at the transcriptionally inactive gene loci. The H1.5-bound intergenic regions did not overlap with the defined enhancer elements [19] in IMR90 cells or with the CCCTC-binding factor (CTCF) binding sites (GSM935404) (data not shown). The H1.5-bound genes function mainly in cell-cell communication and/or response to the environment. Bulk of these genes is expressed in a tissue-specific manner and has evolved in multicellular organisms [20]–[23]. Binding of H1.5 to these loci is established very late in the cellular differentiation process, suggesting that H1.5 may contribute to a terminally differentiated phenotype. H1.5 is an integral member of a larger chromatin regulation system that involves SIRT1 and H3K9me2. This system could establish a stable chromatin state resembling condensed heterochromatin in terminally differentiated cells. H1.5 is, in fact, preferentially located in heterochromatic regions of the genome by immunofluorescence of whole nuclei and has a longer residence time in chromatin compared to other linker histone subtypes [24]. Thus, H1.5 may contribute to the ‘more closed’ chromatin structure in differentiated cells compared to ESCs [17]. Consistent with this, depletion of H1.5 resulted in less chromatin compaction at a target gene family locus. In particular, H1.5 knockdown in fibroblasts resulted in decreased cell growth, which revealed that the function of H1.5 could not be replaced by other linker histone subtypes.

The mechanism by which H1.5 recognizes its target regions at a specific developmental stage is an important question that remains to be answered. While the H1.5 and SIRT1 binding patterns are drastically different in hESCs versus fibroblasts, the levels of both proteins are comparable in the two cell types (Figure S8A), suggesting that mechanisms other than control of expression contribute to H1.5 and SIRT1 binding at gene family loci. These mechanisms may include developmentally-restricted interactions with other chromatin-binding proteins and/or post-translational modifications [25]–[27]. Certain DNA sequence elements and/or chromatin features of the gene family regions may also contribute to H1.5 binding. Considering the tissue specific binding of H1.5 to certain gene families, it is likely that more than one mechanism regulates its genomic distribution.

The binding of H1.5 to the OR genes constitutes a potential mechanism by which the expression of this important gene family cluster could possibly be regulated in olfactory neurons. In each olfactory neuron, only one of the several hundred human OR genes is expressed [28], which may not only involve deliberate activation of a single OR promoter but also active repression of all other OR genes. A recent study reported that OR genes are marked in a highly dynamic fashion by activating and repressive histone modifications in the mouse olfactory epithelium [29]. It will be interesting to study if and how H1.5 may also contribute to the regulation of OR gene expression in olfactory neurons.

Experimental evidence from cancer, ESCs, induced pluripotent cells (iPS) and virally-transformed cells suggest that chromatin states are dynamic and may be perturbed in disease conditions [30]–[32]. Consistent with this, H1.5 expression is down-regulated during cellular transformation by a viral oncoprotein [33] and in many cancer cell types (Figure S8B). In addition, one nonsense (K27*) and one point mutation (G86A) in H1.5 have been reported in colon cancer [8]. The nonsense mutation occurs early in the N-terminus of the protein, essentially eliminating the protein. The G86A mutation is located in the third helix of the conserved globular domain that participates in binding one side of the DNA approximately one helical turn away from the nucleosome dyad [34]. The G86A mutation may thus change the local hydrophilicity and affect the interaction between histone H1.5 and DNA. These data suggest that H1.5 may be down-regulated and/or redistributed during processes that reverse the terminally differentiated state.

## Materials and Methods

### Cell culturing, purification, and differentiation

H1 hESCs were plated on Matrigel (BD Biosciences)-coated plates, and maintained in mTeSR (StemCell). Before purification, cells were trypsinized to single cells and TRA-1-60 expressing cells were isolated by using MACS cell separation columns (Miltenyi Biotec). Isolated cells were tested by flow cytometry, and samples with >99% purity were used. IMR90 human primary lung embryo fibroblasts (ATCC) were cultured in Dulbecco's modified Eagle's medium (DMEM) plus 10% FBS (Hyclone), 100 U/ml penicillin (Gibco), and 100 µg/ml streptomycin (Gibco) at 37°C in 5% CO_2_. Growing cells (passage<8) at 50∼70% confluence were used for further analysis. Human primary hepatocytes (Zen-bio #HP-F) were grown in Hepatocyte Maintenance Medium (Zen-Bio #HM-2) and used at passage four. Human ESCs (HSF1) were differentiated to neural progenitor cells (NPCs) in DMEM:F12 (Gibco) plus B27 (Gibco), N2-supplement (Gibco), 20 ng/ml bFGF (R and D systems), 1 µM Retinoic Acid (Sigma), and 1 µM Smoothened Agonist (Calbiochem). NPCs were mechanically isolated from culture based on rosette morphology as described [35] and expanded in DMEM:F12 plus B27, N2-supplement, 20 ng/ml bFGF, and 50 ng/ml EGF (Gibco). NPCs were further differentiated to neurons and glia by withdrawal of the maintenance factors (bFGF and EGF) for 10 days. Human keratinocytes were cultured per manufacturer's protocol in KSFM (Invitrogen). To induce differentiation, calcium chloride was added to 1.5 mM for 48 hours [36].

### ChIP–chip assay with Agilent promoter array and data analysis

ChIP was performed with ∼50 million cells as described [33]. Agilent two-color microarray data processing is described in Text S1. H1.5 ChIP-chip in IMR90 cells, H1 hESCs, SIRT1^KD^ cells, neural progenitor cells, SIRT1 ChIP-chip in IMR90 cells, H1.5^KD^ cells, and H3K9me2 in IMR90 cells were performed twice. Antibodies against human histone H1.3 (ab24174), H1.5 (ab24175) and SIRT1 (ab32441) were purchased from Abcam and for H3K9me2 from Millipore (07-441).

### ChIP–sequencing assay

∼20 ng of ChIP and input DNA were end-repaired, added an ‘A’ base to the 3′ end, and ligated to adaptors by using Illumina ChIP-seq DNA Sample Preparation Kit Box 1. DNA fragments (150–300 bp) were selected and purified by agarose gel extraction, and amplified by PCR using Phusion polymerase (Illumina ChIP-seq DNA Sample Prep. Kit Box 1) according to manufacturer's instructions. Amplified DNA were purified by gel extraction and quantified by Qubit dsDNA BR assay (Invitrogen). DNA sequencing was performed by Illumina GA-IIx sequencer with read length of 76 bp as per manufacturer's protocol. Raw reads were generated by the software SCS2.6. Further information on data analysis is available via Text S1.

### Whole-genome expression profiling (Affymetrix array) and data analysis

∼200 ng of total RNA were extracted from ∼50 million cells by using Trizol (Invitrogen) and purified by RNeasy Plus Mini Kit (QiaGen). Purified RNA was submitted to UCLA Clinical Microarray Core to perform gene expression profiling by using Affymetrix Human U133Plus2.0 Arrays. Gene expression profiling of IMR90 cells, H1 cells, control^KD^ and H1.5^KD^ cells were performed twice. Probe intensities from different samples were normalized by MAS5.0 provided by Affymetrix.

### mRNA–seq assay

5 µg of total RNA from control^KD^ and H1.5^KD^ IMR90 fibroblasts were used to prepare libraries for mRNA-seq by using mRNA-seq Sample Preparation Kit (Illumina). Sequencing was performed by Illumina HiSeq2000 sequencer with read length of 100 bp as per manufacturer's protocol. Raw reads were aligned to reference human genome (hg19) using TopHat, and expression levels of each gene were calculated by in house software. Detailed data processing information is described in Text S1.

### ChIP–quantitative PCR

Real-time PCR was performed on ChIP and input DNA using SYBR Green Real-time PCR Master Mix (Roche). For each primer pair, an amplification standard curve was established by gradient amount of input DNA. Specific targets were amplified from 1/10 of ChIP DNA, and relevant template DNA amount was calculated by comparing the Ct values of ChIP and input samples to the standard curve.

### Co-immunuprecipitation (co-IP) and Western assay

∼100 million cells were harvested and co-IP assay was performed by using nuclear complex co-IP kit (Active Motif) according to manufacturer's instructions. The precipitates were separated in 4–20% gradient SDS-PAGE gel, and visualized by standard Western blotting assay.

### RNAi assay

siRNAs targeting H1.5 (MU-012049-00) or SIRT1 (MU-003540-01) were purchased from Dharmacon. 1.5 µg of siRNAs were transfected to 2 million cells by using Lipofectamine RNAiMAX (Invitrogen Cat # 13778075). Cells were collected 48 hours after transfection for ChIP-chip, expression microarray, and Western blotting assay.

### Micrococcal nuclease assay

5 million IMR90 cells after control^KD^, H1.5^KD^ or SIRT1^KD^ were trypsinized, pelleted at 4°C for 10 minutes, and washed twice with DPBS. MNase digestion was performed as described [37], [38]. Digested DNA was purified by QiaQuick PCR purification Kit (QiaGen) and quantified by Qubit dsDNA BR assay (Invitrogen). Same amount of DNA was loaded onto a 1.5% agarose gel and run at 50 V overnight at 4°C followed by Ethidium Bromide staining. The gel image was processed by MatLab Image Processing Toolbox.

### Southern blotting

*OR5AS1* probe was prepared by amplifying *OR5AS1* gene DNA with primers 5′-ATGGCTTATGACCGCTATGC and 5′-TTGACGATATTGGAGCCACA from IMR90 genomic DNA. Primers for LCE4A probe are 5′- TGTCCCTCAAAGTGTGCATC and 5′- TTCGCCCACTAATTCCTTTG, and primers for *HIST2H2AA3* probe are 5′- ATTGCCTGGGGTAGTGAGTG and 5′-GCCTTCGTCTTTGAGACTGG. The expected PCR product was gel purified. Biotin labeling was performed by using BrightStar Psoralen-Biotin Nonisotopic Labeling Kit (Ambion AM1480) according to manufacturer's instructions. MNase digested genomic DNA were separated in 1.5% agarose gel, transferred to nylon membrane (Amersham), and cross-linked by UV light. Hybridization was performed by incubating the membrane with labeled probes in Express Hyb Hybridization Solution (Clontech #636831) overnight at 42°C, and signals were detected by using BrightStar BioDetect Kit (Ambion AM1930).

### DNase I assay

5 million IMR90 cells after control^KD^, H1.5^KD^ or SIRT1^KD^ were trypsinized, pelleted at 4°C for 10 minutes, and washed twice with DPBS. DNase I (Roche 04716728001) digestion was performed as described [39]. Digested DNA was purified by phenol/chloroform extraction followed by ethanol precipitation. DNA pellet was air dried and re-suspended in 1× TE buffer. Quantitative PCR solution (20 µL) was prepared by mixing 4 ng of DNA, 20 pmol and 10 µL of FastStart Universal SYBR Green Master (Roche 04913850001), and the reaction was performed by STRATAGENE Mx3000P Real-time PCR machine.

## Supporting Information

Figure S1 Data representation and antibody specificity in ChIP. (A) Pie chart of H1.5 target genes classified by H1.5 enrichment pattern. (B) Design of scaling windows of each gene in ChIP-chip data analysis. Each row in the heat map represents the promoter of a gene in 500-bp intervals from −5.5 to +2.5 kb of the predicted transcriptional start site (TSS). The genes are sorted in descending order based on the average H1.5 promoter enrichment in IMR90 cells. All subsequent heat maps are in the same order. (C) Genomewide promoter distribution of H1.5 in control^KD^ and H1.5^KD^ IMR90 fibroblasts. (D) Localization of H1.3 and H1.5 on gene promoters along chromosomes 1 and 11 in IMR90 fibroblasts. (E) Genomewide promoter distribution of histone H3 in H1 hESCs and IMR90 fibroblasts.
(TIF)

Figure S2 Validation of ChIP-chip data by quantitative ChIP-PCR. ChIP-qPCR of H1.5 and SIRT1 at the LCE4A, LCE1C, SPRR2A, OR5W2, OR5AS1, and HIST3H2A genes at promoter (PRO), transcription start site (TSS), and open reading frame (ORF) regions. Error bars represent the standard deviation of three independent ChIP-qPCR experiments. H1.5 and SIRT1 enrichment were higher in IMR90 (red lines) compared to H1 hESCs (blue lines) at its target genes (LCE4A, LCE1C, SPRR2A, OR5W2, OR5AS1) but not at HIST3H2A which is a gene family member that is not targeted by H1.5.
(TIF)

Figure S3 Expression of hepatocyte specific genes in primary hepatocytes. Relative expression of hepatocyte specific genes in human hepatocytes to IMR90 fibroblasts was calculated from Agilent expression array data. Bars represent the logarithm of the ratio expression in hepatocytes versus IMR90 fibroblasts.
(TIF)

Figure S4 H1.5 binding is associated with gene repression. (A–C) Boxplots of expression levels of randomly selected genes (left) and H1.5 target genes (right) in H1 hESCs, hepatocytes, and calcium induced keratinocytes (HK Ca+). (D) Reciprocal co-immunoprecipitation of SIRT1 and H1.5 from nuclear extracts in IMR90 fibroblasts but not from hESCs.
(TIF)

Figure S5 H1.5 binding is not associated with common histone modifications. (A) Venn diagram of overlaping peaks between H1.5 and indicated histone modifications. (B) Average binding profiles of indicated histone modifications across H1.5 peaks center. (C) Average binding profiles of H1.5 across the peak center of indicated histone modifications.
(TIF)

Figure S6 H1.5 is required for normal cell growth. (A) Expression of linker histone subtype H1.1–H1.5 in knockdown cells by Western blotting. (B) Gene ontology of up- and down-regulated genes in H1.5 knockdown cells. (C) Morphology (left panel), growth curve (line chart), and cell cycle distribution (stacked bar chart) of control^KD^, H1.5^KD^, and SIRT1^KD^ IMR90 cells.
(TIF)

Figure S7 Micrococcal nuclease (MNase) digestion of chromatin. Ethidium bromide staining of MNase treated genomic DNA in control^KD^ (Ctrl), SIRT1^KD^ and H1.5^KD^ IMR90 fibroblasts (A) or H1 hESCs (B). Quantitated data from lanes 5, 10 and 15 (highest MNase concentration) are shown as line chart. Y axis represents the pixel position in the images; x axis shows the band intensity. (C) Patterns of DNase I hypersensitive sites, H3K9me3, H3K27me3 and H1.5 enrichments at representative genes. The scale of DNase I hypersensitive sites represent z-score of counts in each 100-bp window. Scales of H3K9me3, H3K27me3 and H1.5 represent the Poisson p-values of enrichment at each 100-bp window.
(TIF)

Figure S8 H1.5 is generally down-regulated in cancer cells. (A) Expression levels of H1.5 and SIRT1 proteins are similar in hESC and IMR90 fibroblasts as determined by Western blotting. (B) mRNA levels of H1.5 in 173 normal cell types and 744 cancer cell lines from NextBio database [40] are represented as boxplots.
(TIF)

Text S1 Supporting information including additional experimental procedures for data processing, list of gene families in cluster 1 shown in Figure 3, and list of primers used in Figure S2.
(DOC)
