# Supplementary figures and images for: Dynamic Distribution of Linker Histone H1.5 in Cellular Differentiation
Source: PLoS Genet. 2012 Aug 30;8(8):e1002879. doi: 10.1371/journal.pgen.1002879 (PMC3431313; doi:10.1371/journal.pgen.1002879)

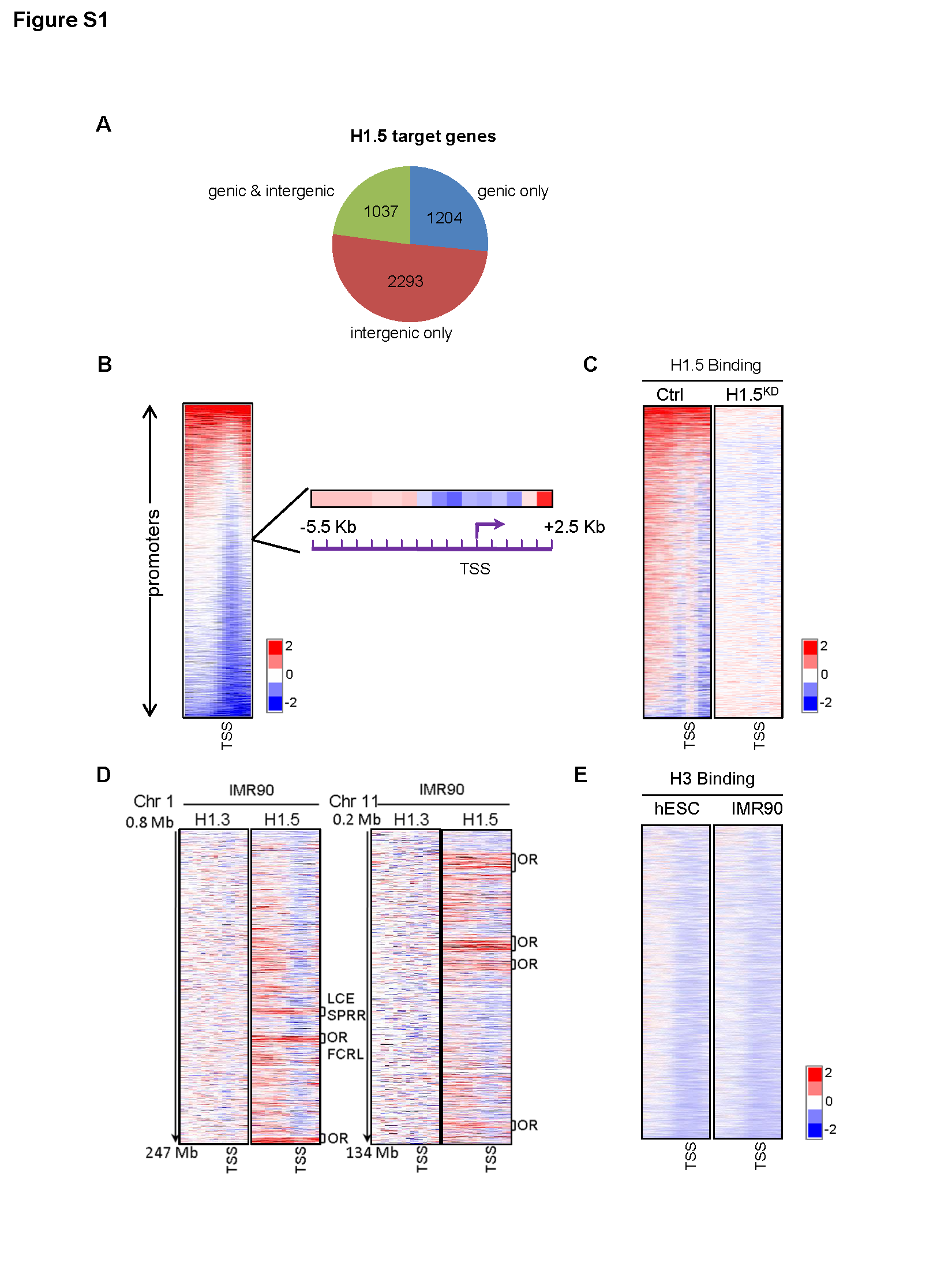

Supplement: Figure S1 — Data representation and antibody specificity in ChIP. (A) Pie chart of H1.5 target genes classified by H1.5 enrichment pattern. (B) Design of scaling windows of each gene in ChIP-chip data analysis. Each row in the heat map represents the promoter of a gene in 500-bp intervals from −5.5 to +2.5 kb of the predicted transcriptional start site (TSS). The genes are sorted in descending order based on the average H1.5 promoter enrichment in IMR90 cells. All subsequent heat maps are in the same order. (C) Genomewide promoter distribution of H1.5 in controlKD and H1.5KD IMR90 fibroblasts. (D) Localization of H1.3 and H1.5 on gene promoters along chromosomes 1 and 11 in IMR90 fibroblasts. (E) Genomewide promoter distribution of histone H3 in H1 hESCs and IMR90 fibroblasts. (TIF) [file pgen.1002879.s001.tif]

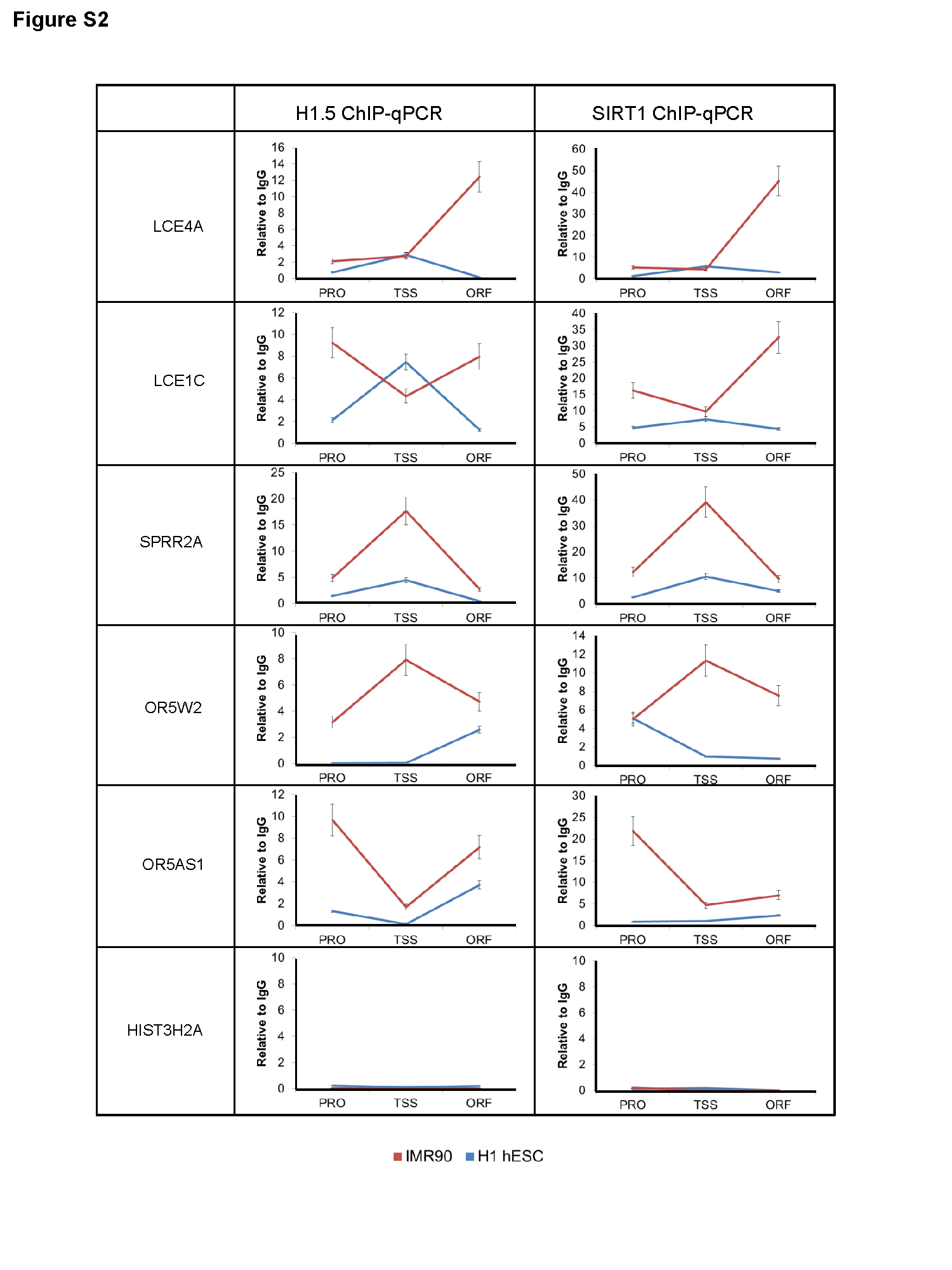

Supplement: Figure S2 — Validation of ChIP-chip data by quantitative ChIP-PCR. ChIP-qPCR of H1.5 and SIRT1 at the LCE4A, LCE1C, SPRR2A, OR5W2, OR5AS1, and HIST3H2A genes at promoter (PRO), transcription start site (TSS), and open reading frame (ORF) regions. Error bars represent the standard deviation of three independent ChIP-qPCR experiments. H1.5 and SIRT1 enrichment were higher in IMR90 (red lines) compared to H1 hESCs (blue lines) at its target genes (LCE4A, LCE1C, SPRR2A, OR5W2, OR5AS1) but not at HIST3H2A which is a gene family member that is not targeted by H1.5. (TIF) [file pgen.1002879.s002.tif]

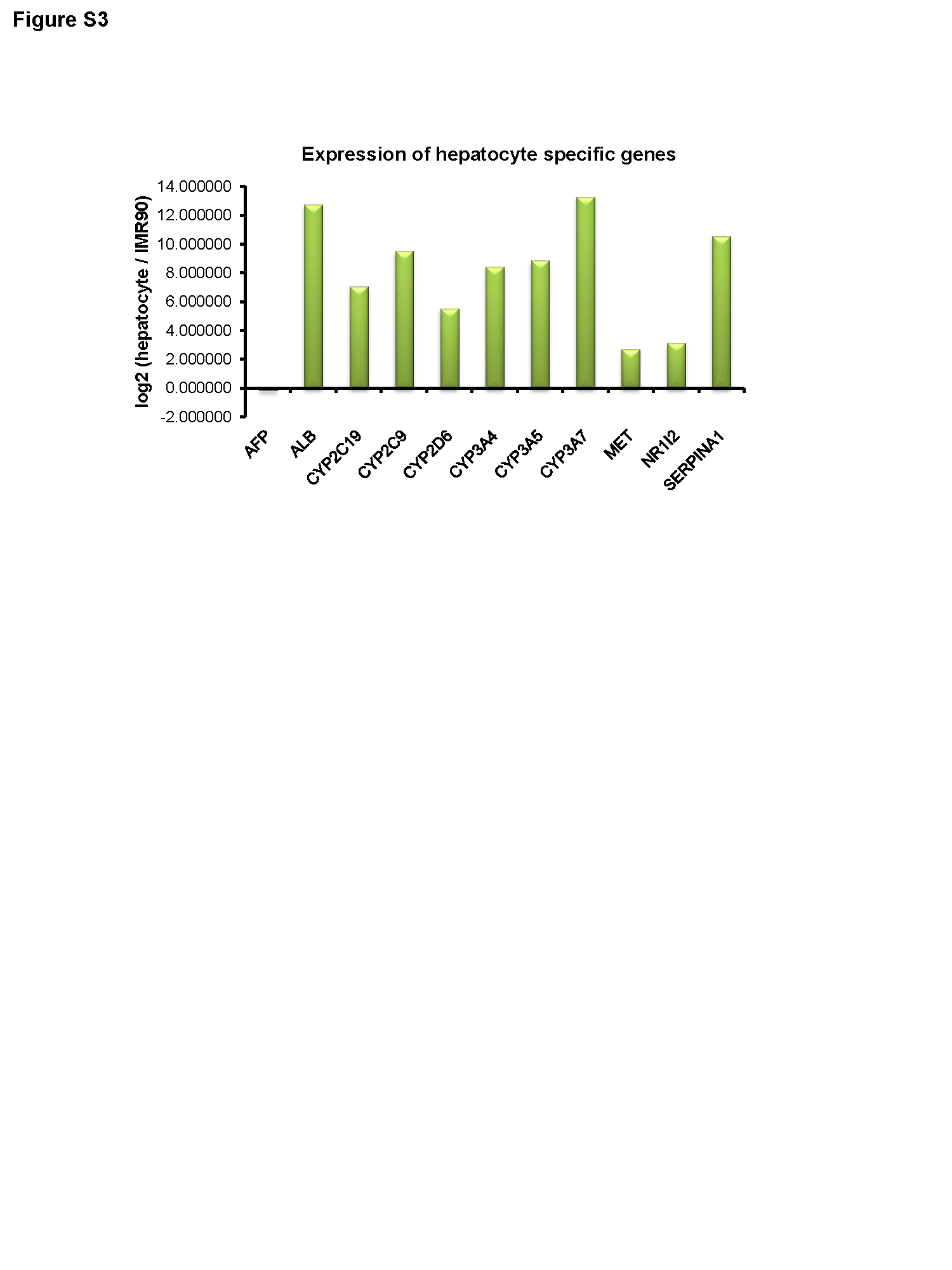

Supplement: Figure S3 — Expression of hepatocyte specific genes in primary hepatocytes. Relative expression of hepatocyte specific genes in human hepatocytes to IMR90 fibroblasts was calculated from Agilent expression array data. Bars represent the logarithm of the ratio expression in hepatocytes versus IMR90 fibroblasts. (TIF) [file pgen.1002879.s003.tif]

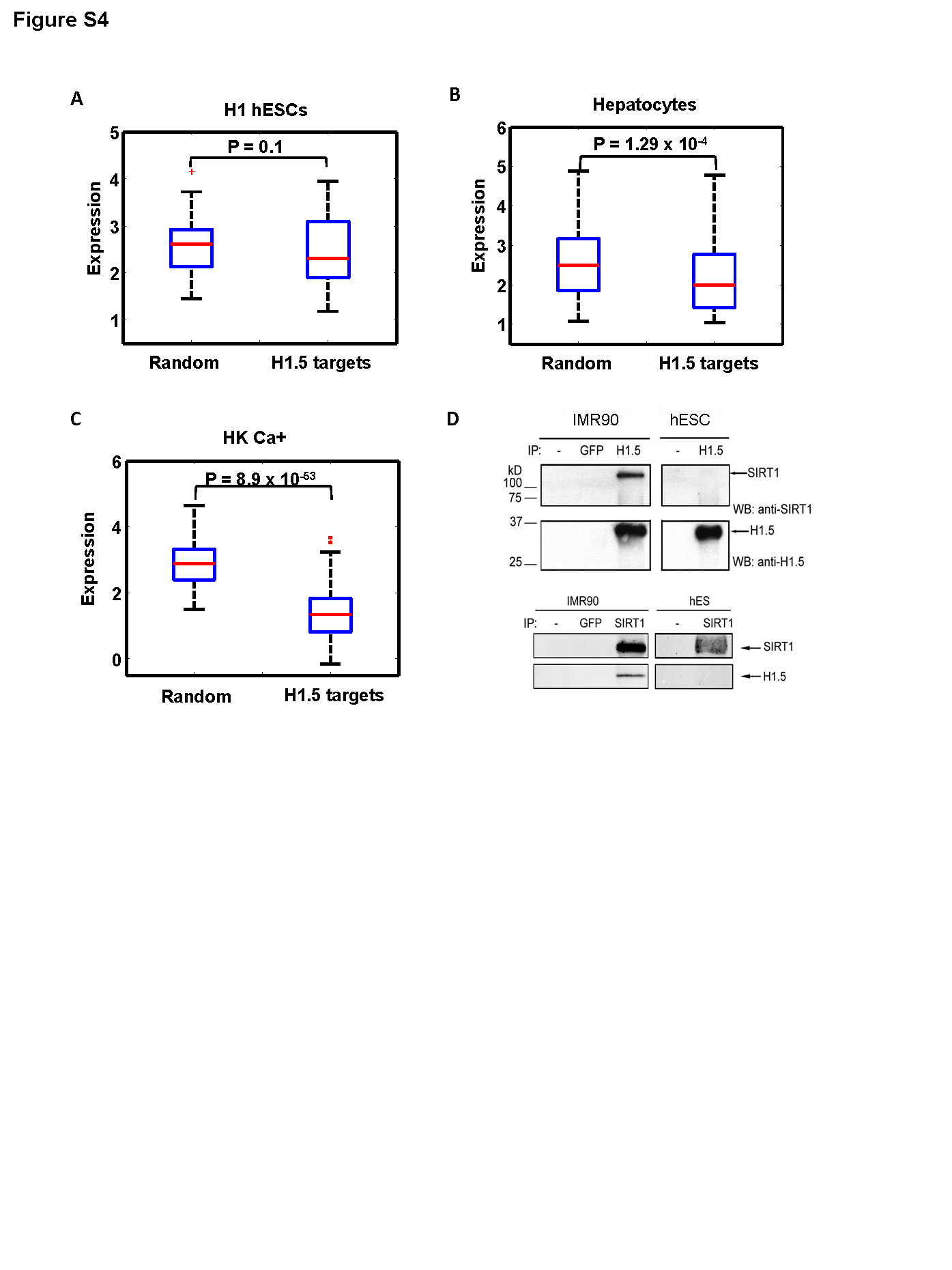

Supplement: Figure S4 — H1.5 binding is associated with gene repression. (A–C) Boxplots of expression levels of randomly selected genes (left) and H1.5 target genes (right) in H1 hESCs, hepatocytes, and calcium induced keratinocytes (HK Ca+). (D) Reciprocal co-immunoprecipitation of SIRT1 and H1.5 from nuclear extracts in IMR90 fibroblasts but not from hESCs. (TIF) [file pgen.1002879.s004.tif]

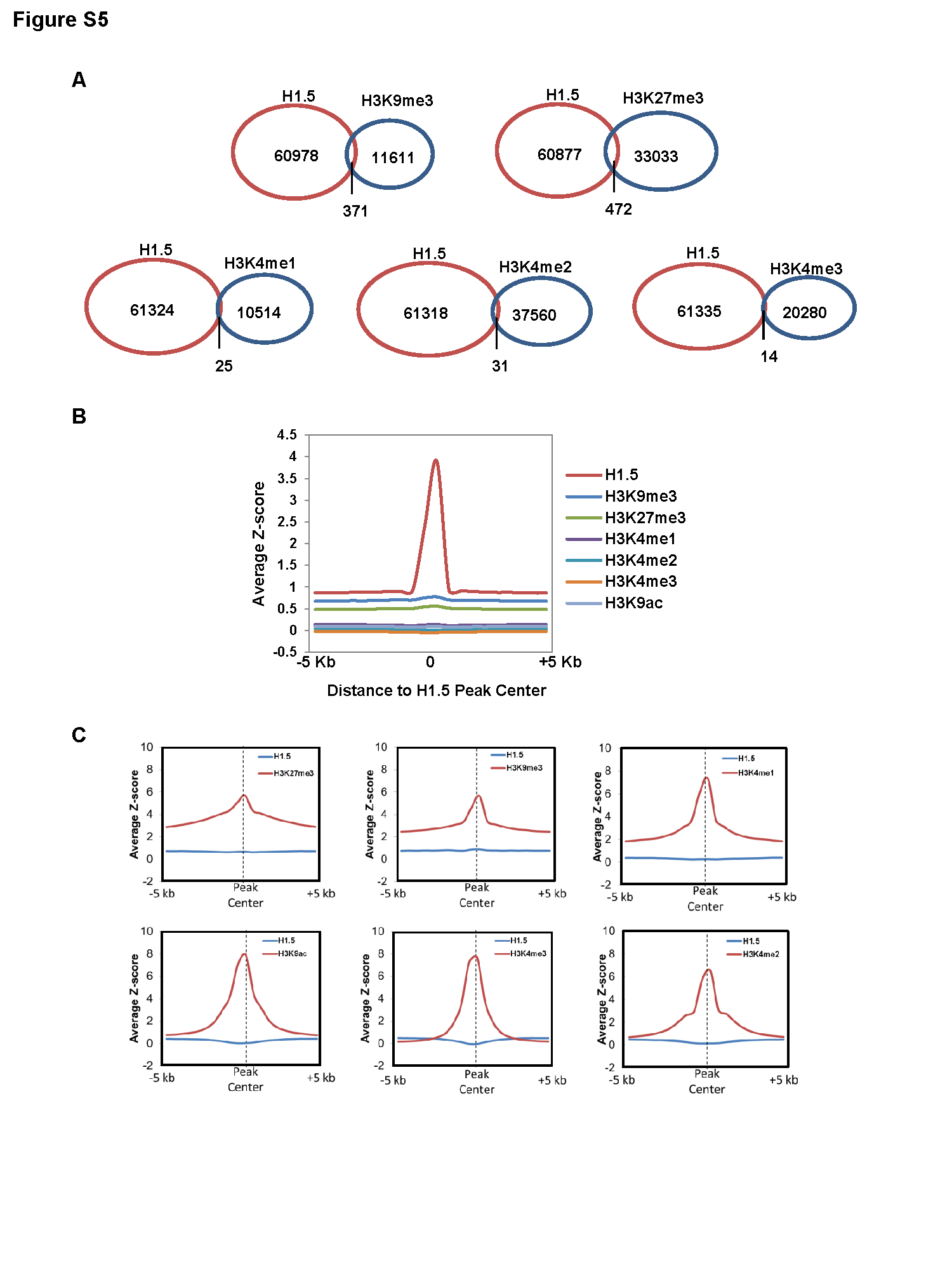

Supplement: Figure S5 — H1.5 binding is not associated with common histone modifications. (A) Venn diagram of overlaping peaks between H1.5 and indicated histone modifications. (B) Average binding profiles of indicated histone modifications across H1.5 peaks center. (C) Average binding profiles of H1.5 across the peak center of indicated histone modifications. (TIF) [file pgen.1002879.s005.tif]

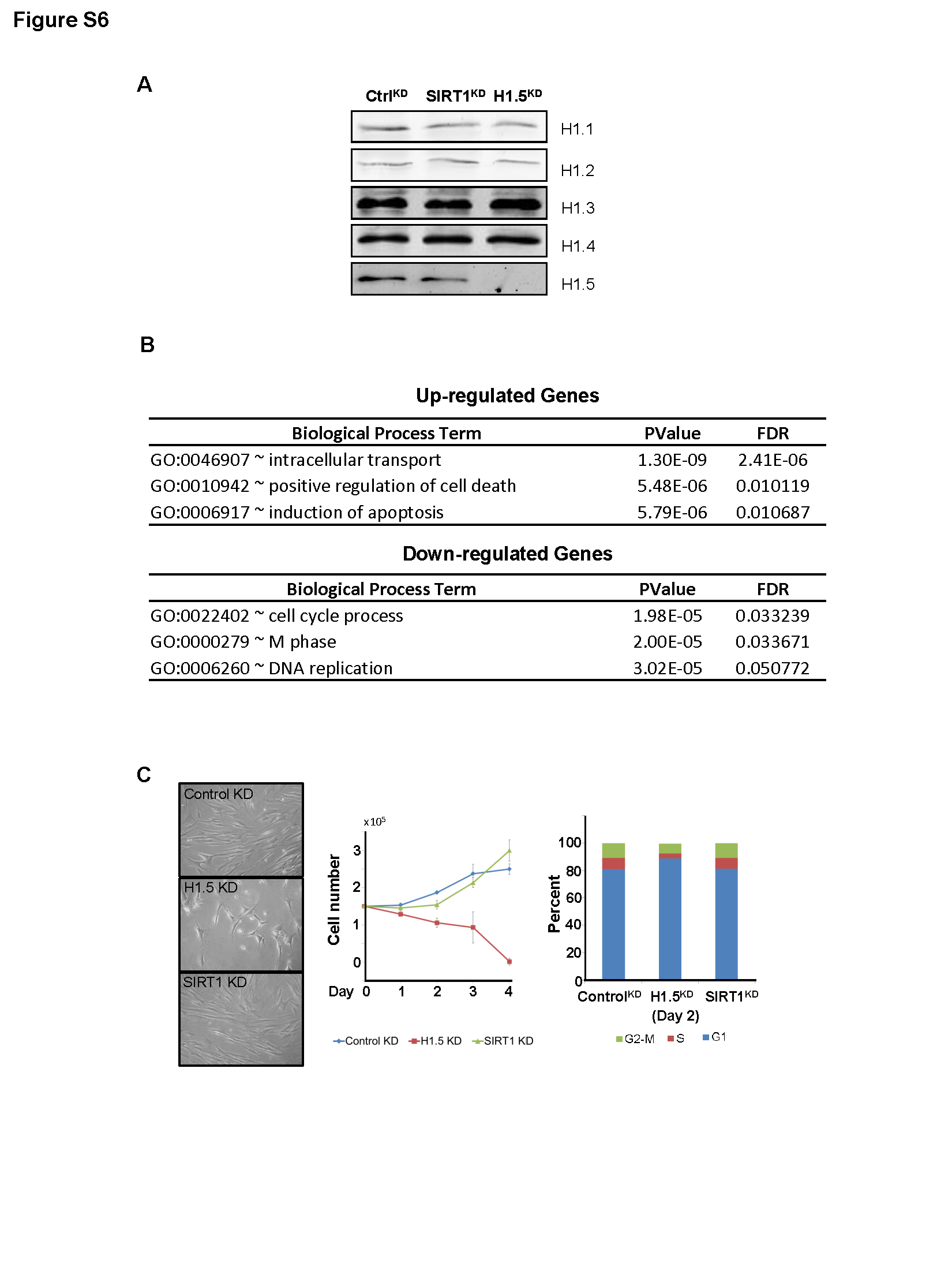

Supplement: Figure S6 — H1.5 is required for normal cell growth. (A) Expression of linker histone subtype H1.1–H1.5 in knockdown cells by Western blotting. (B) Gene ontology of up- and down-regulated genes in H1.5 knockdown cells. (C) Morphology (left panel), growth curve (line chart), and cell cycle distribution (stacked bar chart) of controlKD, H1.5KD, and SIRT1KD IMR90 cells. (TIF) [file pgen.1002879.s006.tif]

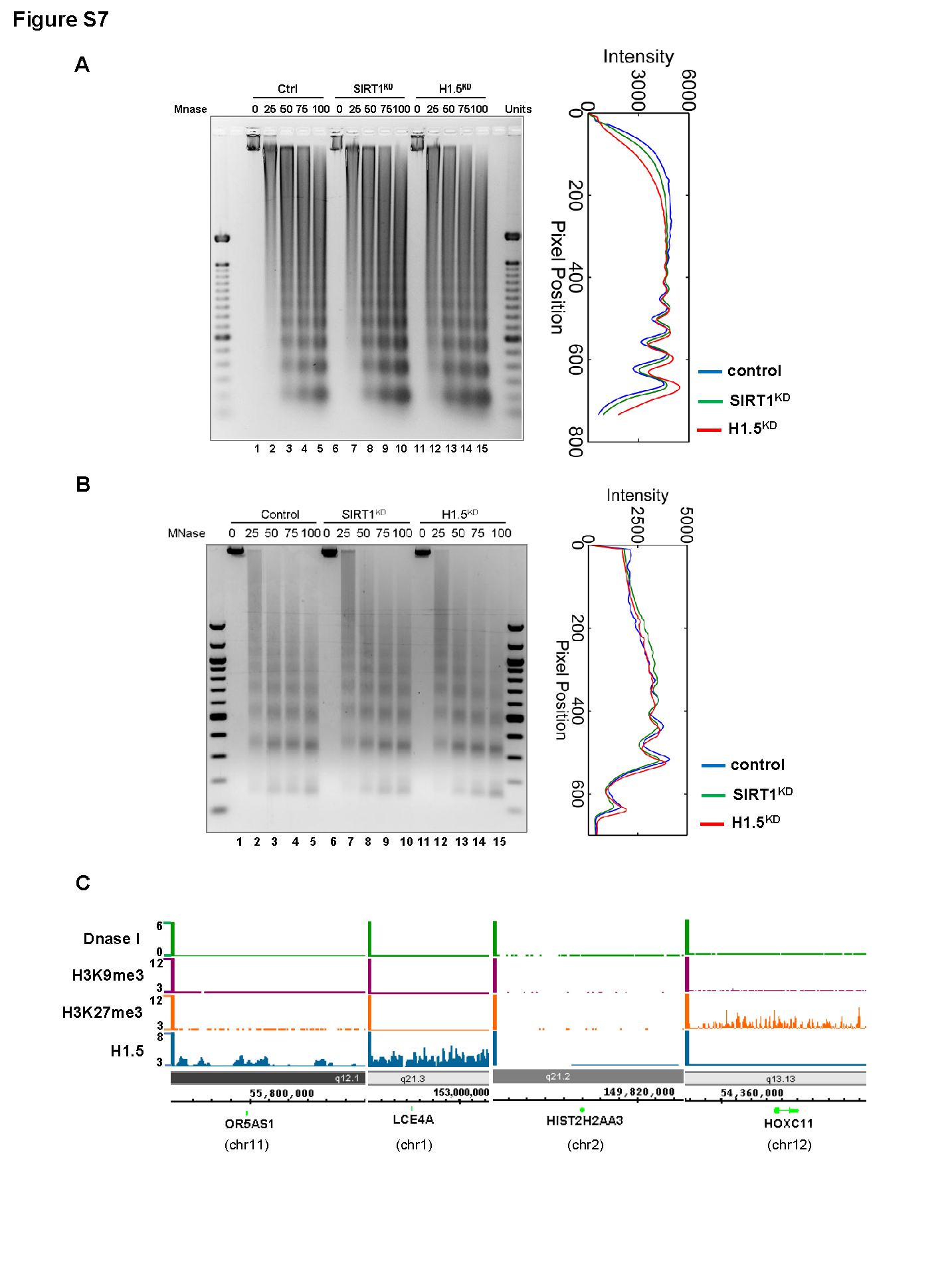

Supplement: Figure S7 — Micrococcal nuclease (MNase) digestion of chromatin. Ethidium bromide staining of MNase treated genomic DNA in controlKD (Ctrl), SIRT1KD and H1.5KD IMR90 fibroblasts (A) or H1 hESCs (B). Quantitated data from lanes 5, 10 and 15 (highest MNase concentration) are shown as line chart. Y axis represents the pixel position in the images; x axis shows the band intensity. (C) Patterns of DNase I hypersensitive sites, H3K9me3, H3K27me3 and H1.5 enrichments at representative genes. The scale of DNase I hypersensitive sites represent z-score of counts in each 100-bp window. Scales of H3K9me3, H3K27me3 and H1.5 represent the Poisson p-values of enrichment at each 100-bp window. (TIF) [file pgen.1002879.s007.tif]

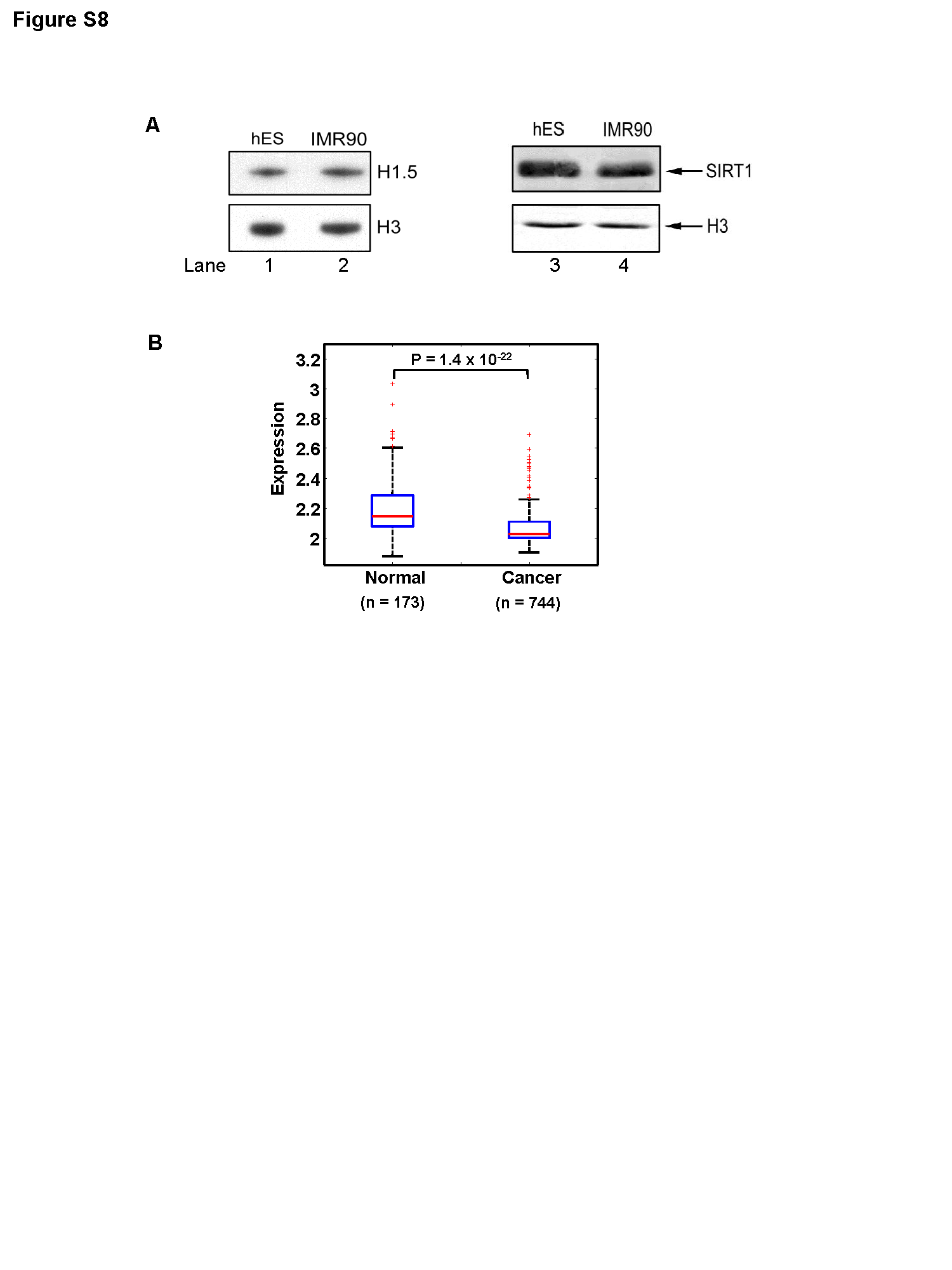

Supplement: Figure S8 — H1.5 is generally down-regulated in cancer cells. (A) Expression levels of H1.5 and SIRT1 proteins are similar in hESC and IMR90 fibroblasts as determined by Western blotting. (B) mRNA levels of H1.5 in 173 normal cell types and 744 cancer cell lines from NextBio database [40] are represented as boxplots. (TIF) [file pgen.1002879.s008.tif]
