# Supplementary material for: Dynamic Distribution of Linker Histone H1.5 in Cellular Differentiation
Source: PLoS Genet. 2012 Aug 30;8(8):e1002879. doi: 10.1371/journal.pgen.1002879 (PMC3431313; doi:10.1371/journal.pgen.1002879)
Supplement: Text S1 — Supporting information including additional experimental procedures for data processing, list of gene families in cluster 1 shown in Figure 3, and list of primers used in Figure S2. (DOC) [file pgen.1002879.s009.doc]

**Text S1**

**Supporting Information**

**Dynamic Distribution of Linker Histone H1.5 in Cellular Differentiation**

Jing-Yu Li, Michaela Patterson, Hanna K.A. Mikkola, William E. Lowry and Siavash K. Kurdistani

**Additional Experimental Procedures**

Data analysis of Agilent Human Promoter ChIP-chip arrays. Data normalization was performed based on the method reported by Marson et al. with some modifications. FeatureExtraction-10.5.1.1 was used to obtain background-subtracted intensity values for each fluorophore. We calculated the median value of blank features and subtracted it from all features on the array. We then calculated the ratio of median values of common features on the array from ChIP and Input channels to generate a normalization factor which was then used to normalize the data. To eliminate probes with low intensities, we divided the background-subtracted intensity values by the background standard deviation values to generate an S factor for each feature on the array. Only features with S > 1.5 on both channels were used for lowess dye bias normalization. To compare data among different samples, logarithm (base 2) ratios of ChIP versus Input in each sample was z-scaled. To visualize and analyse the ChIP-chip data, we divided an 8-kb region from -5.5 to +2.5 kb of the annotated transcriptional start sites (TSS) into 16 fragments of 500 base pairs (bp) each. Average values of normalized features in each windows as well as neighbouring two windows were calculated, sorted based on H1.5 enrichment values of each gene in IMR90 cells and visualized by heat maps (TreeView).

ChIP-seq data analysis. Unique sequences (duplicated sequences were removed and mapped only once to the genome) were aligned to human genome reference HG19 by using Bowtie-0.12.7 (command: bowtie -r -t -o 6 -a -m 1 --best --strata -v 2). Each chromosome was divided into windows of 100 bp. Number of reads in each window was calculated. To normalize total reads of ChIP enriched and Input DNA, the ratio of total reads from ChIP and Input DNA was calculated to generate a normalization factor which was applied to each value of the Input sample. To effectively capture local biases in the genome, we calculated the ratio of ChIP versus Input value in each window, and Poisson distribution was used to calculate a p-value for each window . Significant peaks were defined as enrichment of ChIPed DNA over input DNA within a 100-base pair (bp) window at a Poisson p-value ≤ 0.001. Windows with significant p-values but with no neighboring significant peak or with no input reads were filtered out. To visualize large enrichment blocks, moving average was performed with moving window size of 10 kb and moving steps of 100 bp.

To compare H1.5 binding with other histone modifications, ChIP-seq data of H3K9me3 (GSM521913, GSM521914 and GSM469974), H3K27me3 (GSM521889 and GSM469968), H3K4me1 (GSM521895, GSM521897 and GSM521898), H3K4me2 (GSM521899 and GSM521900), and H3K4me3 (GSM469970 and GSM521901) as well as Input data in IMR90 cells from the Epigenome Roadmap database were processed using the same script as for H1.5 enrichment analysis. When calculating overlapped peaks, each peak was extended 150 bp in both 5’ and 3’ directions. Peaks with at least 1 bp overlap with H1.5 peaks were counted.

For DNase I sequencing data processing, reads in each 100-bp window were counted, and z-scores of counts in each window were calculated. Windows with z-score ≥ 4 were considered as DNase I hypersensitive sites. To Calculate p-value of overlapping, we generated the same number of random peaks as H1.5 peaks, and counted overlapped peaks between random peaks and DNase I hypersensitive sites. This procedure was iterated for 1000 times, and the average number of overlapped peaks was calculated. Binomial p-value of observing the number of overlapped peaks between H1.5 peaks and DNase I hypersensitive sites was calculated.

mRNA-seq data analysis. Unique sequences were aligned to human genome reference HG19 by using TopHat-1.3.2 (default setting). Reads per kb per million reads (RPKM) of each RefSeq genes were calculated for expression comparison. Genes with at least 2 RPKM in either control^KD^ or H1.5^KD^ cells and with at least 1.5 fold changes were considered as changed genes.

**References**

1. Marson A, Kretschmer K, Frampton GM, Jacobsen ES, Polansky JK, et al. (2007) Foxp3 occupancy and regulation of key target genes during T-cell stimulation. Nature 445: 931-935.

2. Zhang Y, Liu T, Meyer CA, Eeckhoute J, Johnson DS, et al. (2008) Model-based analysis of ChIP-Seq (MACS). Genome Biol 9: R137.

Table S1. List of gene families in cluster 1 shown in Figure 3.

| **Family Name** |
| --- |
| ADAM metallopeptidase domain family |
| G protein coupled receptors |
| Cadherin superfamily |
| Immunoglobulins |
| 5 hydroxytryptamine receptors |
| A kinase PRKA anchor protein family |
| Relaxin family peptide receptors |
| Aquaporins |
| Gap junction proteins connexins |
| Keratins |
| Histocompatibility complex |
| C type lectin domain containing |
| Chemokine ligands |
| ATP binding cassette transporters |
| Sodium channels |
| Kallikreins |
| Opsins |
| Axonemal dyneins |
| Collectins |
| Olfactory receptors |
| Anoctamins |
| Interferons |
| Aldo keto reductases |
| Cadherin Superfamily |
| Phosphodiesterases |
| Purinergic receptors |
| Potassium channels |
| Voltage gated ion channels |
| Defensin beta family |
| Defensins alpha family |
| Cytochrome P450 family |
| Mucins |
| UDP glucuronosyltransferases |
| Vomeronasal 1 receptors |
| Sulfotransferase family membrane bound |
| Receptor transporter proteins |
| Nucleotide binding domain and leucine rich repeat containing family |
| Claudins |
| Serine or cysteine peptidase inhibitors |
| Secretoglobins |
| Secreted frizzled related proteins |
| Taste receptors |
| Peptidyl arginine deiminases |
| Membrane associated ring fingers |

List of Primers used in Figure S2.

| Gene | Position | Forwardprimer | Reverse Primer |
| --- | --- | --- | --- |
| *LCE4A* | PRO | TTTGGTGCCAAAGTTCTTCC | CCTTTTCCCAGGCTAAGGTC |
|  | TSS | TGTCCCTCAAAGTGTGCATC | TTCGCCCACTAATTCCTTTG |
|  | ORF | CACCTTGGGGGAGGATTTTA | GGGGATACTTGGGGATAGGA |
| *LCE1C* | PRO | CTGAGCACTCCATCCACTCA | CCTAGGGCTGAGCAACACTC |
|  | TSS | CCACACTGGACAGTTTCAGG | GTTTGGCAAATGCTCTCACA |
|  | ORF | CCTCCTGTCTCTTCCTGCTG | GTCTGTGGCAGTGGGACCTA |
| *SPRR2A* | PRO | GTCCTGGCCAGTGATGATCT | AAGGGGAATGAGGAAGAGGA |
|  | TSS | CCCAGGGTGTCTGAACTTGT | TTTCTGGTTTCTCCCTTCCA |
|  | ORF | AAGGCATGGCTTCTGTGAGT | AGGCAGATCAGTGCTCAGGT |
| *OR5W2* | PRO | GAAGGCAGAGGCTTGAGAGA | TGGCCATTAGATTCCAGAGG |
|  | TSS | TCAACTTCCTTTGGCAGACA | TCCCAGTCCATTCTTCCTTG |
|  | ORF | GCTGTGCTCTGCAATTCTTG | ATAGAGCAGGGGGTTGATGA |
| *OR5AS1* | PRO | TGGAAATTTGCTAGGGTTGG | AAGCCAGCCTTCTTTGTGAA |
|  | TSS | GCAGCGCGTTTAAAGAGGTA | TTGTTTTTCCCACTGGACCT |
|  | ORF | ATGGCTTATGACCGCTATGC | TTGACGATATTGGAGCCACA |
| *HIST3H2A* | PRO | AGGCAGGGATGGAAAAGTCT | CTGGCCCCAAATGTCTTTTA |
|  | TSS | TCTTGCCGTCCTTCTTCTGT | CCAGCGATGACGTAGAACAA |
|  | ORF | AAATGTCCGGTCGTGGTAAG | AGCTTGTTGAGCTCCTCGTC |
